# Supplementary material for: Hot luminescence from single-molecule chromophores electrically and mechanically self-decoupled by tripodal scaffolds
Source: Nat Commun. 2023 Dec 12;14:8253. doi: 10.1038/s41467-023-43948-y (PMC10716191; doi:10.1038/s41467-023-43948-y)
Supplement: Supplementary file 1 — Supplementary Information [file 41467_2023_43948_MOESM1_ESM.pdf]

Supplementary Information for

Hot luminescence from single-molecule chromophores electrically and mechanically self-decoupled by tripodal scaffolds

Vibhuti Rai[1 ‡], Nico Balzer[2 ‡], Gabriel Derenbach[1], Christof Holzer[3], Marcel Mayor[1, 2, 4, 5, *], Wulf Wulfhekel[1, 6], Lukas Gerhard[1, *], and Michal Valášek[2, *]

[1] Institute for Quantum Materials and Technologies, Karlsruhe Institute of Technology (KIT), Kaiserstr. 12, 76131 Karlsruhe, Germany

[2] Institute of Nanotechnology, Karlsruhe Institute of Technology (KIT), Kaiserstr. 12, 76131 Karlsruhe, Germany

[3] Institute of Theoretical Solid State Physics, Karlsruhe Institute of Technology (KIT), Kaiserstr. 12, 76131 Karlsruhe, Germany

[4] Department of Chemistry, University of Basel, St. Johannsring 19, CH-4056 Basel, Switzerland

[5] Lehn Institute of Functional Materials, Sun Yat-Sen University (SYSU), Xingang West Road, Guangzhou, China

[6] Physikalisches Institut, Karlsruhe Institute of Technology (KIT), Kaiserstr. 12, 76131 Karlsruhe, Germany

‡ These authors contributed equally to this work

* Email: marcel.mayor@unibas.ch, lukas.gerhard@kit.edu, michal.valasek@kit.edu

Table of Contents

Supplementary Methods 3

Supplementary Note 1. Surface Deposition of Tol-Tpd-sNDI-Ac and Tol-Tpd-nNDI-Ac Molecules 10

Supplementary Note 2. Light Collection and STML Measurements 10

Supplementary Note 3. Reproducibility of the STML Spectrum on different Tol-Tpd Molecules 11

Supplementary Note 4. Bias-dependent STML Measurements on Tol-Tpd-nNDI 12

Supplementary Note 5. Current-dependent STML Measurements 13

Supplementary Note 6. Time-dependent Density Functional Theory Investigations 14

Supplementary Note 7. Spectrophotometric Studies of Tol-Tpd-sNDI-Ac and Tol-Tpd-nNDI-Ac 19

Supplementary Note 8. NMR Spectra of all new Compounds 20

Supplementary References 30

Supplementary Methods

3,3,3-Tris(4-bromophenyl)methanol 1

This compound was synthesized using a modified literature procedure[1]. Under argon, 1,4-dibromobenzene (16.1 g, 68.3 mmol, 3.9 eq) was dissolved in dry THF (270 mL). After cooling down to -78 °C, *n*-BuLi (39.7 mL, 63.6 mmol, 3.7 eq) was added dropwise. After 10 min, the solution turned into a white suspension and was stirred for another 1 h at -78 °C. Subsequently, diethyl carbonate (2.1 mL, 17.3 mmol, 1 eq) was added dropwise over 15 min and the reaction mixture was allowed to warm to room temperature and stirred overnight. After quenching the reaction with NH_4Cl (10% aq., 50 mL), the aqueous phase was extracted with ethyl acetate (3 × 50.0 mL) and the combined organic layer was washed with brine (50 mL) and dried over $MgSO_4$. After filtration and evaporation of all volatiles, the crude product was purified by flash chromatography on silica gel to afford 7.80 g (92%) of the title compound as a white solid. R_f = 0.27 (hexane/EtOAc 8:1); m.p. 106 °C (decomposition); ^{1}H NMR (500 MHz, $CDCl_3$) δ (ppm) = 7.42 (d, J = 8.7 Hz, 6H, $C^{3,5}H$), 7.10 (d, J = 8.7 Hz, 6H, $C^{2,6}H$), 2.78 (s, 1H, OH); ^{13}C NMR (126 MHz, $CDCl_3$) δ (ppm) = 145.0 (C^1), 131.5 ($C^{3,5}H$), 129.7 ($C^{2,6}H$), 122.1 (C^4), 81.3 (C-OH); FTIR (ATR): $\tilde{\nu}$ (cm^{-1}) = 3408 (m, v(OH)), 1589 (w), 1572 (w), 1485 (m), 1393 (m), 1317 (m), 1297 (w), 1270 (w), 1177 (w), 1150 (m, $v(R_3C\text{-}O)$), 1073 (m), 1010 (m), 1000 (m), 947 (w), 918 (w), 907 (m), 809 (m), 588 (w), 580 (w), 519 (w), 476 (w); MS (EI, 70 eV) *m/z* (%): 497.9 (3) $[M]^+$, 341.0 (23), 185.0 (100), 157.0 (38), 76.0 (54).

3,3,3-Tris(4-bromophenyl)methyl chloride 2

An oven dried 60 mL pressure tube was charged with 3,3,3-tris(4-bromophenyl)methanol **1** (1.00 g, 2.01 mmol, 1.0 eq.) and acetyl chloride (1.5 mL, 21.14 mmol, 10.5 eq.), 40 mL dry toluene, and sealed. Then, the mixture was heated up to 120 °C and was stirred for 16 h at this temperature. After cooling down to room temperature, the solvent, the residue acetyl chloride and acetic acid were removed under reduced pressure and suspended in anhydrous hexane (50 mL). Subsequent filtration and vacuum drying yielded the target compound **2** as a white solid (1.04 g) in quantitative yield. The obtained product was used for the next step without further purification. ^{1}H NMR (500 MHz, $CDCl_3$) δ (ppm) = 7.42 (d, J = 8.7 Hz, 6H, $C^{3,5}H$), 7.06 (d, J = 8.7 Hz, 6H, $C^{2,6}H$); ^{13}C NMR (126 MHz, $CDCl_3$) δ (ppm) = 143.6 (C^1), 131.4 ($C^{3,5}H$), 131.3 ($C^{2,6}H$), 122.8 (C^4), 79.5 (C-Cl); MS (EI, 70 eV) *m/z* (%): 478.9 (20) $[M-Cl]^+$, 445.0 (18), 368.9 (12), 319.0 (12), 239.1 (50), 182.9 (100), 119.6 (55), 76.0 (20).

3-[3,3,3-Tris(4-bromophenyl)]-1-trimethylsilylpropyne 3

Trimethylsilylethynylmagnesium bromide solution: An oven dried Schlenk flask was charged with trimethylsilylacetylene (1.07 mL, 7.5 mmol, 1.25 eq.) and 6 mL anhydrous tetrahydrofuran

under an argon atmosphere. The resulting mixture was cooled down to 0 °C and ethylmagnesium bromide (2.00 mL, 6.0 mmol, 1.00 eq.; 3 M in diethyl ether) was dropwise added. The milky suspension was stirred for 15 min until no further gas evolution was observed. The reaction mixture was allowed to warm to room temperature and then heated to 60 °C and stirred for 1 h, whereby it turned into a brown-orange solution. This freshly prepared Grignard reagent was allowed to cool to room temperature and used directly in the next step without further purification.

In a Schlenk flask, trityl chloride **2** (1.01 g, 1.96 mmol, 1.0 eq.) was dissolved in dry toluene (50 mL) and the resulting solution was heated up to 60 °C. After reaching this temperature, the freshly prepared trimethylsilylethynylmagnesium bromide in tetrahydrofuran (0.68 mL, 6.00 mmol, 3.0 eq.) was added dropwise and the reaction mixture was stirred overnight. After cooling down to room temperature, the reaction was quenched with ammonium chloride solution (20 mL, 10 % aq.) and the aqueous phase was extracted with ethyl acetate (3 × 20 mL) The combined organic layer was washed with brine and dried over $MgSO_4$. The solvent was removed under reduced pressure and the crude product was purified by column chromatography on silica gel in hexane. The title compound was obtained as a white solid (877 mg) in 78% yield. R_f = 0.66 (hexane); m.p. 132 °C; ^{1}H NMR (500 MHz, $CDCl_3$) δ (ppm) = 7.40 (dd, *J* = 8.6 Hz, J = 1.8 Hz, 6H, $C^{3,5}H$), 7.06 (dd, *J* = 8.6 Hz, J = 1.8 Hz, 6H, $C^{2,6}H$), 0.19 (s, 9H, CH_3); ^{13}C NMR (126 MHz, $CDCl_3$) δ (ppm) = 143.5 (C^1), 131.5 ($C^{3,5}H$), 130.9 ($C^{2,6}H$), 121.6 (C^4), 110.1 (≡C^{II}-TMS), 90.9 (-C^{I}≡), 55.5 (C), 0.1 (CH_3); FTIR (ATR**)**: $\tilde{\nu}$ (cm^{-1}) = 2960 (w, $\nu_{as}(CH_3)$), 2177 (w), 1484 (m), 1474 (w), 1396 (w), 1246 (w), 1076 (m), 1062 (w), 1010 (m), 843 (m), 814 (m), 758 (w), 528 (w), 496 (w); MS (EI, 70 eV) *m/z* (%): 496.95 (7) $[M-Br]^+$, 423.04 (6), 281.08 (7); 207.06 (30); 73.05 (100) $[TMS]^+$.

3-[2-(Trimethylsilyl)ethylsulfanyl]phenylacetylene 5

In an oven dried Schlenk-flask, compound **4** (2.12 g, 7.3 mmol, 1.0 eq), $Pd(PPh_3)_2Cl_2$ (360 mg, 0.5 mmol, 0.1 eq) and copper(I)-iodide (140 mg, 0.7 mmol, 0.1 eq) were dissolved in anhydrous triethylamine (7 mL) and purged with argon for 10 min. At 70 °C, trimethylsilylacetylene (1.4 mL, 10.3 mmol, 1.4 eq) was added and the reaction mixture was stirred for 3 h at this temperature. The reaction was quenched with NH_4Cl solution (10 % aq., 10 mL), diluted with diethyl ether (30 mL) and then the layers were separated. The organic phase was washed with water (2 × 20 mL), brine (20 mL) and dried with $MgSO_4$. After filtration and removal of all volatiles, the crude product was dissolved in hexane, passed over a pad of silica gel (15 g) and washed with pure hexane (500 mL). The solvent was evaporated in a round bottom flask and the crude product was used for the next step without further purification. The crude product and potassium carbonate (8.0 g) were dispersed in a mixture of DCM (30 mL) and methanol (30 mL) and stirred at room temperature for 1 h. The reaction was slowly neutralized with 2M

HCl. After separation of the organic and the aqueous layer, the aqueous phase was washed with DCM (3 × 50 mL), and the combined organic layer was washed with brine (50 mL) and dried over $MgSO_4$. After filtration and evaporation of all volatiles, the crude product was purified by flash chromatography on silica gel (200 g, hexane) to afford 1.4 g (82%) of the desired compound as a pale-yellow oil. R_f = 0.44 (hexane); ^{1}H NMR (500 MHz, $CDCl_3$) δ (ppm) = 7.38 (s, 1H, C^2H), 7.30 - 7.24 (m, 2H, C^4H, C^6H), 7.22 - 7.19 (m, 1H, C^5H), 3.06 (s, 1H, CH), 3.00 - 2.92 (m, 2H, CH_2-S), 0.92 - 0.88 (m, 2H, CH_2-TMS), 0.03 (s, 9H, CH_3); ^{13}C NMR (126 MHz, $CDCl_3$) δ (ppm) = 138.1 (C^3), 132.0 (C^2H), 129.5 (C^6H), 129.3 (C^4H), 128.9 (C^5H), 122.9 (C^1), 83.4 (-C^I≡), 77.7 (≡C^{II}H), 29.5 (CH_2-S), 16.9 (CH_2-TMS), -1.6 (CH_3); FTIR (ATR): $\tilde{\nu}$ (cm^{-1}) = 3293 (m, *v*(≡CH)), 2952 (m, v_{as}(CH_3)), 2919 (w, v_{as}(CH_2)), 2895 (w), 1585 (w), 1562 (m), 1471 (w), 1398 (w), 1260 (m), 1248 (m), 1198 (w), 1163 (w), 1095 (w), 1082 (w), 1010 (w), 884 (w), 855 (m), 837 (m), 783 (m), 752 (w), 727 (w), 685 (m), 647 (m), 616 (m); MS (EI, 70 eV) *m/z* (%): 234.1 (2) $[M]^+$, 191.1 (11), 73.1 (100).

Compound Tol-Tpd-TMS

Under inert conditions, in an oven dried Schlenk flask, compound **3** (317 mg, 0.5 mmol, 1 eq), $Pd(PPh_3)_4$ (114 mg, 0.1 mmol, 0.2 eq) and copper(I)-iodide (11 mg, 55.0 mmol, 0.10 eq) were dissolved in freshly distilled triethylamine (2 mL) and purged with argon for 15 min. At 80 °C, compound **5** (450 mg, 1.9 mmol, 3.50 eq), dissolved in freshly distilled and degassed triethylamine (1 mL), was added and the reaction mixture was stirred for 4 h at this temperature. The reaction was quenched with NH_4Cl solution (10% aq., 15 mL) and diluted with ethyl acetate (15 mL). The aqueous phase was extracted with ethyl acetate (3 × 5 mL) and the combined organic layer was washed with brine and dried with $MgSO_4$. After filtration and evaporation of all volatiles, the crude product was purified by flash chromatography on silica gel (hexane/DCM 5:1) to yield 439 mg (77%) of the title compound as white solid. R_f = 0.12 (hexane/DCM 5:1); m.p. 122 °C; ^{1}H NMR (500 MHz, $CDCl_3$) δ (ppm) = 7.45 (d, *J* = 8.4 Hz, 6H, $C^{3',5'}$H), 7.42 (s, 3H, C^2H), 7.31 - 7.28 (m, 3H, C^6H), 7.26 - 7.23 (m, 6H, C^4H, C^5H), 7.22 (d, *J* = 8.4 Hz, 6H, $C^{2',6'}$H), 2.97 - 2.94 (m, 6H, CH_2-S), 0.94 - 0.90 (m, 6H, CH_2-TMS), 0.21 (s, 9H, CH_3), 0.03 (s, 27H, CH_3); ^{13}C NMR (126 MHz, $CDCl_3$) δ (ppm) = 144.9 ($C^{1'}$), 138.0 (C^3), 131.6 ($C^{3',5'}$H), 131.5 (C^2H), 129.3 ($C^{2',6'}$H), 129.0 (C^4H), 128.9 (C^5H), 128.8 (C^6H), 124.0 (C^1), 122.2 ($C^{4'}$), 110.5 (-C^I≡), 90.9 (≡C^{II}-TMS), 89.60 (≡C^{IV}-), 89.55 (-C^{III}≡), 56.4 (C), 29.5 (CH_2-S), 16.9 (CH_2-TMS), 0.1 (CH_3, TMS), -1.5 (CH_3, TMSE); FTIR (ATR): $\tilde{\nu}$ (cm^{-1}) = 2951 (m, v_{as}(CH_3)), 2921 (m, v_{as}(CH_2)), 2852 (m, v_{sym}(CH_2)), 2168 (w), 2163 (w), 1584 (m), 1560 (w), 1502 (m), 1470 (w), 1439 (w), 1404 (w), 1259 (m), 1248 (m), 1163 (w), 1147 (w), 1094 (w), 1079 (w), 1062 (w), 1019 (w), 854 (m), 825 (m), 781 (m), 758 (m), 749 (m), 726 (w), 684 (m); ESI (+) HRMS calcd for $C_{63}H_{72}S_3Si_4K$: 1075.3510 $[M + K]^+$, found *m/z* 1075.3460.

Compound Tol-Tpd-H

In a round bottom flask compound **Tol-Tpd**-TMS (439 mg, 0.4 mmol, 1 eq) and potassium carbonate (584 mg, 4.2 mmol, 10 eq) were dispersed in the mixture of THF (10 mL) and methanol (10 mL) and stirred for 3 h. Then, the reaction mixture was diluted with ethyl acetate (10 mL) and slowly neutralized with 2 M HCl. The aqueous phase was extracted with ethyl acetate (3 × 20 mL) and the combined organic layer was washed with brine (30 mL) and dried with $MgSO_4$. After filtration and evaporation of all volatiles, the crude product was purified by flash chromatography on silica gel (200 g, hexane/ethyl acetate 60:1) to afford 398 mg (97%) of the desired compound as a pale-yellow oil. R_f = 0.40 (hexane/ethyl acetate 60:1); ^{1}H NMR (500 MHz, $CDCl_3$) δ (ppm) = 7.46 (d, J = 8.5 Hz, 6H, $C^{3',5'}H$), 7.43 (s, 3H, C^2H), 7.31 - 7.28 (m, 3H, C^6H), 7.26 - 7.23 (m, 12H, C^4H, C^5H, $C^{2',6'}H$), 2.98 - 2.95 (m, 6H, CH_2-S), 2.76 (s, 1H, ≡C^{II}-H), 0.94 - 0.91 (m, 6H, CH_2-TMS), 0.04 (s, 27H, CH_3); ^{13}C NMR (126 MHz, $CDCl_3$) δ (ppm) = 144.4 ($C^{1'}$), 138.0 (C^3), 131.7 ($C^{3',5'}H$), 131.5 (C^2H), 129.3 ($C^{2',6'}H$), 129.0 (C^4H), 128.92 (C^5H), 128.87 (C^6H), 124.0 (C^1), 122.3 ($C^{4'}$), 89.7 (≡C^{IV}-), 89.5 (-C^{III}≡), 88.6 (-C^I≡), 74.7 (≡C^{II}-H), 55.5 (C), 29.5 (CH_2-S), 16.9 (CH_2-TMS), -1.5 (CH_3); FTIR (ATR**)**: $\tilde{\nu}$ (cm^{-1}) = 3296 (w, ν(≡CH)), 2950 (m, $\nu_{as}(CH_3)$), 2919 (m, $\nu_{as}(CH_2)$), 2895 (w) 2852 (w, $\nu_{sym}(CH_2)$), 1583 (m), 1560 (m), 1501 (m), 1471 (w), 1439 (w), 1403 (m), 1259 (m), 1247 (m), 1162 (w), 1147 (w), 1094 (w), 1080 (w), 1019 (w), 887 (w), 854 (m), 824 (s), 780 (m), 749 (m), 726 (w), 684 (m), 661 (w), 641 (w), 551 (w); ESI (+) HRMS calcd for $C_{60}H_{64}S_3Si_3$: 964.3478 $[M]^+$, found *m/z* 964.3465.

Compound Tol-Tpd-sNDI-TMSE

Under inert conditions, in an oven dried Schlenk-flask, **sNDI**-I (132 mg, 138 µmol, 1.0 eq), $Pd(PPh_3)_4$ (8 mg, 7 µmol, 0.05 eq) and copper(I)-iodide (3 mg, 14 µmol, 0.1 eq) were dissolved in dry THF (2 mL) and purged with argon for 30 min. Then compound **Tol-Tpd**-H (140 mg, 145 µmol, 1.05 eq) dissolved in freshly distilled NEt_3 (4 mL) was added and the reaction mixture was stirred overnight at room temperature. The reaction mixture was quenched with NH_4Cl solution (10%, 5 mL) and diluted with ethyl acetate (15 mL). The aqueous phase was extracted with ethyl acetate (3 × 5 mL) and the combined organic layer was washed with brine and dried with $MgSO_4$. After filtration and evaporation of all volatiles, the crude product was purified by flash chromatography on silica gel (120 g, hexane/DCM 2:3, R_f = 0.40). After drying under vacuum, 182 mg (74%) of the desired compound **Tol-Tpd-sNDI**-TMSE was isolated as a red solid. R_f = 0.40 (hexane/DCM 2:3); m.p. 227 °C; ^{1}H NMR (500 MHz, $CDCl_3$) δ (ppm) = 8.03 (s, 1H, C^3H, NDI), 8.00 (s, 1H, C^7H, NDI), 7.67 (d, J = 8.4 Hz, 2H, $C^{3'',5''}H$), 7.50 (m, 1H, $C^{4'''}H$), 7.49 (d, J = 8.5 Hz, 6H, $C^{3',5'}H$), 7.44 (s, 3H, C^2H), 7.35 - 7.28 (m, 11H, $C^{2'',6''}H$, $C^{2',6'}H$, C^6H), 7.26 - 7.23 (m, 6H, $C^{4,5}H$), 7.07 (d, J = 1.6 Hz, 2H, $C^{2''',6'''}H$), 7.05 (d, J = 5.4 Hz, 4H, $C^{3^{IV},5^{IV}}H$), 2.99 - 2.95 (m, 6H, CH_2-S), 2.34 (s, 12H, *o*-CH_3), 2.30 (s, 6H, *p*-CH_3), 1.33 (s, 18H, CH_3,

tert-Bu), 0.95 - 0.91 (m, 6H, CH_2-TMS), 0.04 (s, 27H, CH_3, TMS); ^{13}C NMR (126 MHz, $CDCl_3$) δ (ppm) = 163.9 ($\underline{C}$O-C^5), 163.7 ($\underline{C}$O-C^1), 162.7 (2×$\underline{C}$O-C^{4,8}), 152.2 (C$^{3''',5'''}$), 149.9 (C^6, NDI), 149.3 (C^2, NDI), 144.8 (C$^{1'}$), 143.8 (C$^{2IV, 6IV}$), 141.24 (C^{4IV}), 141.19 (C^{4IV}), 138.0 (C^3), 134.8 (C$^{1''}$), 134.2 (C$^{1'''}$), 132.9 (C$^{3'',5''}$H), 131.8 (C$^{3',5'}$H), 131.5 (C^{2}H), 130.4 (C$^{3IV, 5IV}$H), 129.5 (C^{3,7}H, NDI), 129.3 (C$^{2',6'}$H), 129.0 (C$^{2'',6''}$H), 128.91 (C^{4,5}H), 128.86 (C^{6}H), 126.43 (C^5, NDI), 126.39 (C^1, NDI), 125.4 (C^4, NDI), 125.1 (C^{1IV}), 125.0 (C^{1IV}), 124.3 (C$^{4''}$), 124.1 (C^8, NDI), 124.0 (C^1), 123.7 (C$^{4'''}$H), 122.5 (C$^{2''',6'''}$H), 122.3 (C$^{4'}$), 119.9 (C^{8a}, NDI), 119.0 (C^{4a}, NDI), 95.6 (-C^{I}≡), 89.7 (≡C^{IV}-), 89.5 (-C^{III}≡), 85.5 (≡C^{II}-), 56.1 (C, core), 35.2 (C, *tert*-Bu), 31.7 (CH_3, *tert*-Bu), 29.5 ($\underline{C}H_2$-S), 21.8 (*o*-CH_3), 21.51 (*p*-CH_3), 21.49 (*p*-CH_3), 16.9 ($\underline{C}H_2$-TMS), -1.5 (CH_3, TMS); FTIR (ATR): $\tilde{\nu}$ (cm^{-1}) = 2953 (m, $\upsilon_{as}(CH_3)$), 2920 (m), 2866 (w, $\upsilon_{sym}(CH_3)$), 1705 (m, υ(C=O)), 1665 (m), 1584 (w), 1551 (m), 1504 (m), 1431 (m), 1321 (m), 1247 (m), 1224 (m), 1165 (m), 1019 (w), 825 (m), 785 (m), 749 (m), 684 (w); UV-Vis (DCM): λ_{max} (nm) (ε (mol^{-1}dm^{3}cm^{-1})) = 295 (144400), 356 (13596), 370 (14327), 523 (24610); ESI (+) HRMS calcd for $C_{112}H_{112}N_2O_4S_5Si_3Na$: 1815.6431 [M + Na]$^+$, found *m/z* 1815.6422.

Compound Tol-Tpd-nNDI-TMSE

Under inert conditions, in an oven dried Schlenk flask, **nNDI**-I (105 mg, 132 µmol, 1.0 eq), $Pd(PPh_3)_4$ (8 mg, 7 µmol, 0.05 eq) and copper(I)-iodide (3 mg, 13 µmol, 0.1 eq) were dissolved in dry THF (2 mL) and purged with argon for 30 min. Then compound **Tol-Tpd**-H (140 mg, 145 µmol, 1.1 eq) dissolved in freshly distilled NEt_3 (4 mL) was added and the reaction mixture was stirred overnight at room temperature. The reaction mixture was quenched with NH_4Cl solution (10%, 5 mL) and diluted with ethyl acetate (15 mL). The aqueous phase was extracted with ethyl acetate (3 × 5 mL) and the combined organic layer was washed with brine and dried with $MgSO_4$. After filtration and evaporation of all volatiles, the crude product was purified by flash chromatography on silica gel (100 g, hexane/DCM 3:1). After drying, 94 mg (71%) of the desired compound **Tol-Tpd-nNDI**-TMSE was isolated as a blue solid. R_f = 0.40 (hexane/DCM 3:1); m.p. 133 °C; ^{1}H NMR (500 MHz, $CDCl_3$) δ (ppm) = 8.42 (s, 1H, C^{3}H, NDI), 8.41 (s, 1H, C^{7}H, NDI), 7.70 (d, *J* = 8.4 Hz, 2H, C$^{3'',5''}$H), 7.51 (m, 1H, C$^{4'''}$H), 7.50 (dd, *J* = 8.5 Hz, *J* = 1.7 Hz, 6H, C$^{3',5'}$H), 7.44 (d, *J* = 1.0 Hz, 3H, C^{2}H), 7.36 - 7.27 (m, 11H, C$^{2'',6''}$H, C$^{2',6'}$H, C^{6}H), 7.26 - 7.22 (m, 6H, C^{4,5}H), 7.09 (d, *J* = 1.6 Hz, 2H, C$^{2''',6'''}$H), 3.48 (d, *J* = 5.1 Hz, 8H, C^{2IV,5IV}H_2), 2.99 - 2.94 (m, 6H, CH_2-S), 2.01 (m, 8H, C^{3IV,4IV}H_2), 1.35 (s, 18H, CH_3, *tert*-Bu), 0.96 - 0.90 (m, 6H, CH_2-TMS), 0.04 (s, 27H, CH_3, TMS); ^{13}C NMR (126 MHz, $CDCl_3$) δ (ppm) = 164.1 ($\underline{C}$O-C^4), 164.0 ($\underline{C}$O-C^8), 162.2 (C^6, NDI), 161.8 (C^2, NDI), 151.8 (C$^{3''',5'''}$), 147.9 ($\underline{C}$O-C^5), 147.7 ($\underline{C}$O-C^1), 144.9 (C$^{1'}$), 138.0 (C^3), 136.4 (C$^{1''}$), 135.4 (C$^{1'''}$), 132.9 (C$^{3'',5''}$H), 131.8 (C$^{3',5'}$H), 131.5 (C^{2}H), 129.3 (C$^{2',6'}$H), 129.2 (C$^{2'',6''}$H), 129.0 (C^{6}H), 128.91 (C^{4}H), 128.86 (C^{5}H), 125.8 (C^4, NDI), 125.1 (C^8, NDI), 124.0 (C^1), 123.7 (C$^{4''}$), 123.2 (C^{1,5}, NDI), 123.1 (C$^{2''',6'''}$H), 122.9 (C$^{4'''}$H), 122.3 (C$^{4'}$), 122.2 (C^{3}H, NDI), 122.1 (C^{7}H, NDI), 106.0 (C^{8a}, NDI), 105.2 (C^{4a}, NDI), 95.2 (-C^{I}≡), 89.7

(- C^{III}≡), 89.6 (≡C^{IV}-), 85.9 (≡C^{II}-), 56.1 (C, core), 52.8 ($C^{2IV,5IV}H_2$), 35.2 (C, *tert*-Bu), 31.7 (CH_3, *tert*-Bu), 29.5 ($\underline{C}H_2$-S), 26.1 ($C^{3IV,4IV}H_2$), 16.9 ($\underline{C}H_2$-TMS), -1.5 (CH_3, TMS); FTIR (ATR**)**: $\tilde{\nu}$ (cm^{-1}) = 2951 (m, $\upsilon_{as}(CH_3)$), 2921 (m, $\upsilon_{as}(CH_2)$), 2851 (m, $\upsilon_{sym}(CH_2)$), 1693 (m, υ(C=O)), 1656 (m), 1584 (w), 1567 (m), 1503 (m), 1471 (m), 1448 (m), 1425 (m), 1355 (m), 1341 (w), 1330 (m), 1319 (m), 1259 (w), 1247 (m), 1215 (m), 1161 (w), 1141 (m), 1119 (w), 886 (w), 855 (m), 825 (m), 780 (m), 749 (m), 722 (w), 706 (w), 685 (m); UV-Vis (DCM): λ_{max} (nm) (ε ($mol^{-1}dm^3cm^{-1}$)) = 294 (120879), 348 (11720), 365 (13303), 602 (19441); ESI (+) HRMS calcd for $C_{102}H_{106}N_4O_4S_3Si_3Na$: 1653.6582 $[M + Na]^+$, found *m/z* 1653.6578.

Compound Tol-Tpd-sNDI-Ac

In an oven dried Schlenk flask, compound **Tol-Tpd-sNDI**-TMSE (145 mg, 81 µmol, 1.0 eq) was dissolved in dry DCM (4 mL), cooled down to 0 °C and flushed with argon before acetyl chloride (0.4 mL) was added and the reaction mixture was stirred for 20 min. Then, $AgBF_4$ (110 mg, 566 µmol, 7 eq) was added and the reaction mixture was stirred for another 3 h to reach room temperature. Afterwards, the reaction mixture was quenched with crashed ice, diluted with DCM (5 mL) and extracted with DCM (3 × 5 mL). The combined organic layer was dried with $MgSO_4$. After filtration and evaporation of all volatiles at room temperature, the crude product was purified by flash chromatography on silica gel (100 g, hexane/DCM 1:2). After drying, 81 mg of **Tol-Tpd-sNDI**-Ac was isolated as a red solid in 62% yield. R_f = 0.29 (hexane/DCM 1:2); m.p. 50 °C (decomposition);^{1}H NMR (500 MHz, $CDCl_3$) δ (ppm) = 8.03 (s, 1H, C^3H, NDI), 8.00 (s, 1H, C^7H, NDI), 7.67 (d, *J* = 8.5 Hz, 2H, $C^{3'',5''}$H), 7.59 (s, 3H, C^2H), 7.56 (dt, *J* = 6.9 Hz, *J* = 1.8 Hz, 3H, C^6H), 7.51 (m, 1H, $C^{4'''}$H), 7.49 (d, *J* = 8.5 Hz, 6H, $C^{3',5'}$H), 7.41 - 7.35 (m, 6H, $C^{4,5}$H), 7.33 (d, *J* = 8.4 Hz, 2H, $C^{2'',6''}$H), 7.31 (d, *J* = 8.5 Hz, 6H, $C^{2',6'}$H), 7.07 (d, *J* = 1.7 Hz, 2H, $C^{2''',6'''}$H), 7.05 (d, *J* = 5.4 Hz, 4H, $C^{3IV,5IV}$H), 2.43 (s, 9H, CH_3, Ac), 2.34 (s, 12H, *o*-CH_3), 2.31 (s, 6H, *p*-CH_3), 1.33 (s, 18H, CH_3, *tert*-Bu); ^{13}C NMR (126 MHz, $CDCl_3$) δ (ppm) = 193.7 (CO, Ac), 163.9 ($\underline{C}$O-C^5), 163.7 ($\underline{C}$O-C^1), 162.7 (2×$\underline{C}$O-$C^{4,8}$), 152.2 ($C^{3''',5'''}$), 149.9 (C^6, NDI), 149.3 (C^2, NDI), 144.9 ($C^{1'}$), 143.77 and 143.76 ($C^{2IV,6IV}$), 141.23 (C^{4IV}), 141.19 (C^{4IV}) 137.5 (C^2H), 134.8 ($C^{1''}$), 134.5 (C^4H), 134.2 ($C^{1'''}$), 132.9 ($C^{3'',5''}$H), 132.7 (C^6H), 131.8 ($C^{3',5'}$H), 130.4 ($C^{3IV,5IV}$H), 129.5 (C^3H, NDI), 129.35 (C^5H), 129.33 ($C^{2',6'}$H, $C^{2'',6''}$H), 129.0 (C^7H, NDI), 128.5 (C^3), 126.43 (C^5, NDI), 126.39 (C^1, NDI), 125.3 (C^4, NDI), 125.1 (C^{1IV}), 124.9 (C^{1IV}), 124.6 (C^1), 124.2 ($C^{4''}$), 124.1 (C^8, NDI), 123.7 ($C^{4'''}$H), 122.5 ($C^{2''',6'''}$H), 122.2 ($C^{4'}$), 119.9 (C^{8a}, NDI), 119.0 (C^{4a}, NDI), 95.5 (-C^I≡), 90.3 (-C^{III}≡), 89.0 (≡C^{IV}-), 85.6 (≡C^{II}-), 56.1 (C, core), 35.2 (C, *tert*-Bu), 31.7 (CH_3, *tert*-Bu), 30.5 (CH_3, Ac), 21.8 (*o*-CH_3), 21.51 (*p*-CH_3), 21.49 (*p*-CH_3); FTIR (ATR**)**: $\tilde{\nu}$ (cm^{-1}) = 2952 (m, $\upsilon_{as}(CH_3)$), 2921 (m), 2854 (m, $\upsilon_{sym}(CH_3)$), 1704 (m, υ(C=O)), 1665 (m), 1587 (w), 1550 (m), 1503 (m), 1464 (w), 1431 (m), 1403 (w), 1362 (w), 1322 (m), 1248 (m), 1227 (m), 1166 (w), 1110 (m), 1078 (w), 946 (w), 824 (w), 786 (m), 750 (w), 741 (w), 684 (w), 612

(m), 536 (w); UV-Vis (DCM): λ_{max} (nm) (ε ($mol^{-1}dm^{3}cm^{-1}$)) = 294 (121671), 356 (13027), 370 (13647), 524 (21918); ESI (+) HRMS calcd for $C_{103}H_{82}N_2O_7S_5Na$: 1641.4623 $[M + Na]^+$, found *m/z* 1641.4621; Elemental analysis calcd for $C_{103}H_{82}N_2O_7S_5$: C 76.36, H 5.10, N 1.73, found: C 76.68, H 4.92, N 1.81.

Compound Tol-Tpd-nNDI-Ac

In an oven dried Schlenk flask, the compound **Tol-Tpd-nNDI**-TMSE (64 mg, 39 µmol, 1 eq) was dissolved in dry DCM (3 mL), cooled down to 0 °C and flushed with argon. Then, acetyl chloride (0.3 mL) was added, and the mixture was stirred for 20 min before $AgBF_4$ (38 mg, 196 µmol, 5 eq) was added. The original blue reaction mixture changed the colour to pink and after ≈5 min, to a milky suspension, which was stirred for 5 h to reach room temperature. The reaction mixture was quenched with crashed ice and neutralised with sodium hydrogen carbonate to pH ≈ 6. After extraction with DCM, the combined organic layer was dried with $MgSO_4$. After filtration and evaporation of all volatiles at room temperature, the crude product was purified by flash chromatography on silica gel (80 g, hexane/DCM 1:4). After drying, 43 mg (76%) of the title compound **Tol-Tpd-nNDI**-Ac was isolated as a blue solid. R_f = 0.12 (hexane/DCM 1:4); m.p. 50 °C (decomposition); ^{1}H NMR (500 MHz, $CDCl_3$) δ (ppm) = 8.42 (s, 1H, $C^{3}H$, NDI), 8.41 (s, 1H, $C^{7}H$, NDI), 7.70 (d, *J* = 8.4 Hz, 2H, $C^{3'',5''}H$), 7.59 (s, 3H, $C^{2}H$), 7.56 (dt, *J* = 7.0 Hz, *J* = 1.7 Hz, 3H, $C^{6}H$), 7.52 (t, *J* = 1.7 Hz, 1H, $C^{4'''}H$), 7.49 (d, *J* = 8.5 Hz, 6H, $C^{3',5'}H$), 7.41 - 7.35 (m, 6H, $C^{4,5}H$), 7.33 (d, *J* = 8.5 Hz, 6H, $C^{2',6'}H$), 7.31 (d, *J* = 8.4 Hz, 2H, $C^{2'',6''}H$), 7.10 (d, *J* = 1.6 Hz, 2H, $C^{2''',6'''}H$), 3.47 (d, *J* = 5.0 Hz, 8H, $C^{2IV,5IV}H_2$), 2.42 (s, 9H, CH_3, Ac), 2.01 (m, 8H, $C^{3IV,4IV}H_2$), 1.36 (s, 18H, CH_3, *tert*-Bu); ^{13}C NMR (126 MHz, $CDCl_3$) δ (ppm) = 193.7 (CO, Ac), 164.1 (<u>C</u>O-C^{4}), 164.0 (<u>C</u>O-C^{8}), 162.2 (C^{6}, NDI), 161.8 (C^{2}, NDI), 151.8 ($C^{3''',5'''}$), 147.8 (<u>C</u>O-C^{5}), 147.6 (<u>C</u>O-C^{1}), 145.0 ($C^{1'}$), 137.5 ($C^{2}H$), 136.3 ($C^{1''}$), 135.4 ($C^{1'''}$), 134.5 ($C^{4}H$), 132.8 ($C^{3'',5''}H$), 132.7 ($C^{6}H$), 131.8 ($C^{3',5'}H$), 129.3 ($C^{2',6'}H$, $C^{2'',6''}H$), 129.2 ($C^{5}H$), 128.5 (C^{3}), 125.7 (C^{4}, NDI), 125.1 (C^{8}, NDI), 124.6 (C^{1}), 123.7 ($C^{4''}$), 123.2 ($C^{1,5}$, NDI), 123.1 ($C^{2''',6'''}H$), 122.9 ($C^{4'''}H$), 122.2 ($C^{4'}$), 122.1 ($C^{3}H$, $C^{7}H$, NDI), 106.0 (C^{8a}, NDI), 105.3 (C^{4a}, NDI), 95.1 (- C^{I}≡), 90.3 (-C^{III}≡), 89.0 (≡C^{IV}-), 85.9 (≡C^{II}-), 56.1 (C, core), 52.8 ($C^{2IV,5IV}H_2$), 35.2 (C, *tert*-Bu), 31.7 (CH_3, *tert*-Bu), 30.5 (CH_3, Ac), 26.1 ($C^{3IV,4IV}H_2$); FTIR (ATR**)**: $\tilde{\nu}$ (cm^{-1}) = 2953 (m), 2923 (m, υ_{as}(CH_2)), 2855 (m), 1692 (m, υ(C=O)), 1654 (m), 1587 (w), 1566 (m), 1502 (w), 1476 (m), 1448 (m), 1426 (m), 1354 (m), 1342 (w), 1330 (w), 1318 (w), 1213 (m), 1111 (m), 1079 (w), 944 (w), 885 (w), 824 (w), 781 (m), 751 (w), 726 (w), 684 (w), 612 (w); UV-Vis (DCM): λ_{max} (nm) (ε ($mol^{-1}dm^{3}cm^{-1}$)) = 293 (117239), 349 (10031), 365 (11795), 602 (17870); ESI (+) HRMS calcd for $C_{93}H_{76}N_4O_7S_3Na$: 1479.4774 $[M + Na]^+$, found *m/z* 1479.4800; Elemental analysis calcd for $C_{93}H_{76}N_4O_7S_3$: C 76.62, H 5.25, N 3.84, found: C 76.29, H 5.42, N 3.71.

Supplementary Note 1. Surface Deposition of Tol-Tpd-sNDI-Ac and Tol-Tpd-nNDI-Ac Molecules

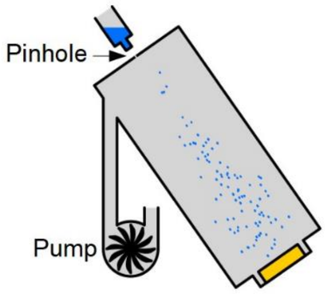

Suppl. Fig. 1 │ Schematic showing the spray deposition setup used for molecules Tol-Tpd-sNDI-Ac **and Tol-Tpd-nNDI**-Ac. Reproduced from Ref.[2] with permission from the Royal Society of Chemistry.

Supplementary Note 2. Light Collection and STML Measurements

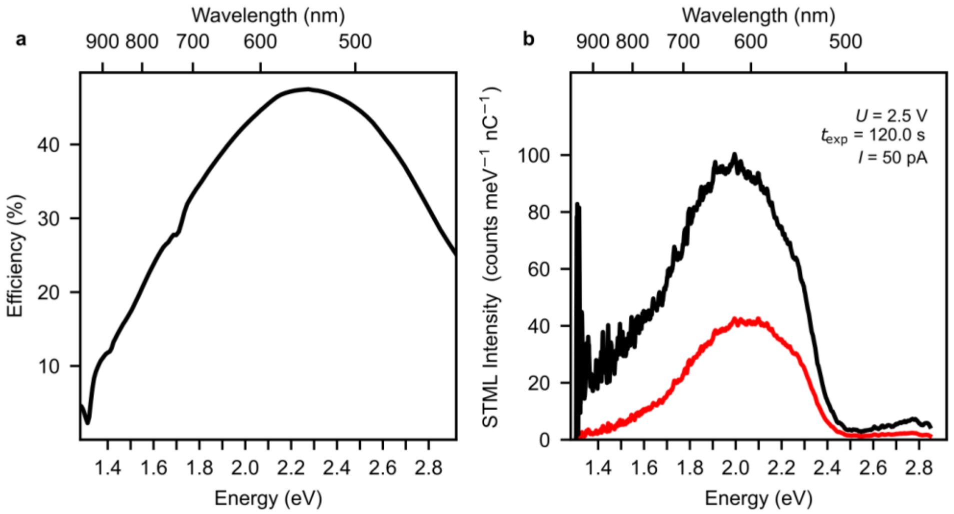

Suppl. Fig. 2 │ Light collection and plasmonic cavity. a Detector efficiency of the light collection setup excluding the microfabricated mirror tip. **b** Plasmonic light emission of the mirror tip used in this work. Red/black lines show the signal uncorrected/corrected for detector efficiency.

Supplementary Note 3. Reproducibility of the STML Spectrum on different Tol-Tpd Molecules

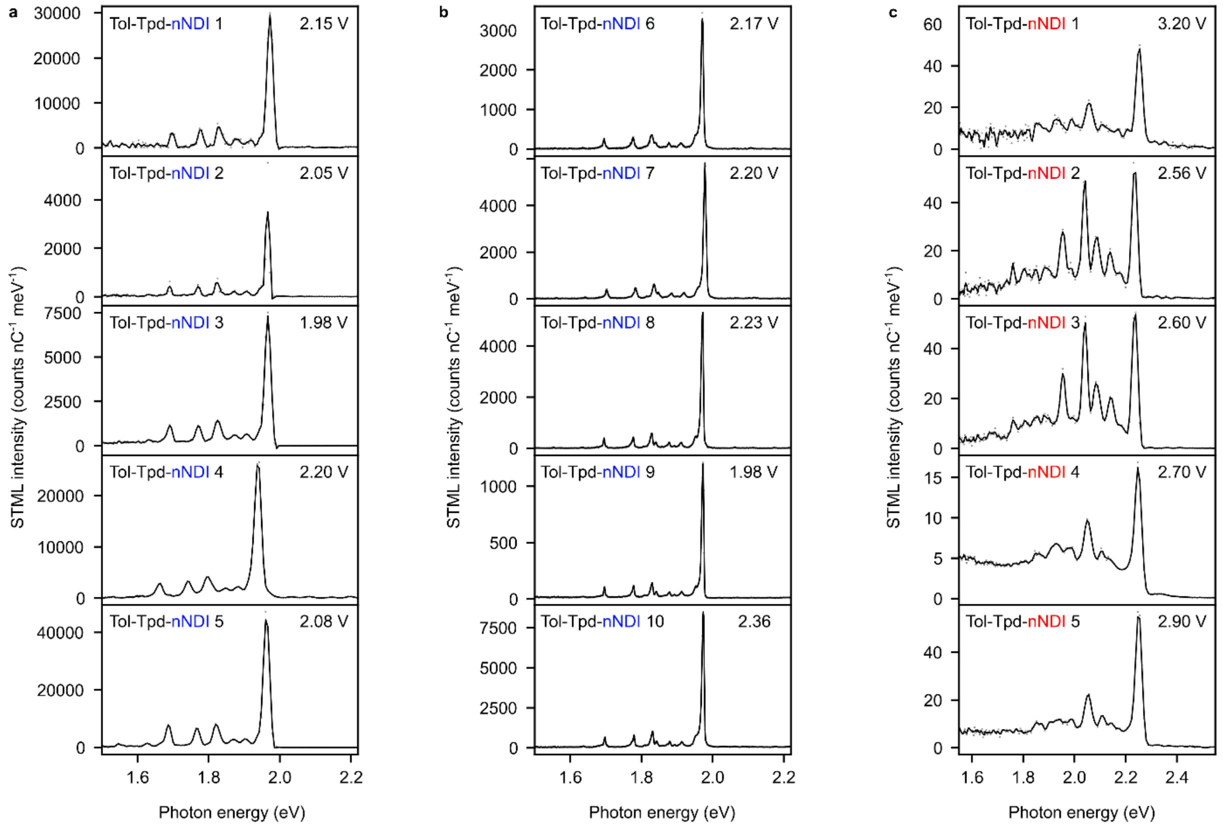

Suppl. Fig. 3 │ STML spectra recorded on ten different individual Tol-Tpd-nNDI molecules and five different Tol-Tpd-sNDI molecules showing their high reproducibility. a Tol-Tpd-nNDI 1-5: spectrometer with entrance slit fully open. **b Tol-Tpd-nNDI** 6-10: spectrometer with entrance slit closed to 10 µm. **c Tol-Tpd-sNDI** 1-5: spectrometer with entrance slit fully open. The applied bias voltage is shown in the upper right of each graph.

Supplementary Note 4. Bias-dependent STML Measurements on Tol-Tpd-nNDI

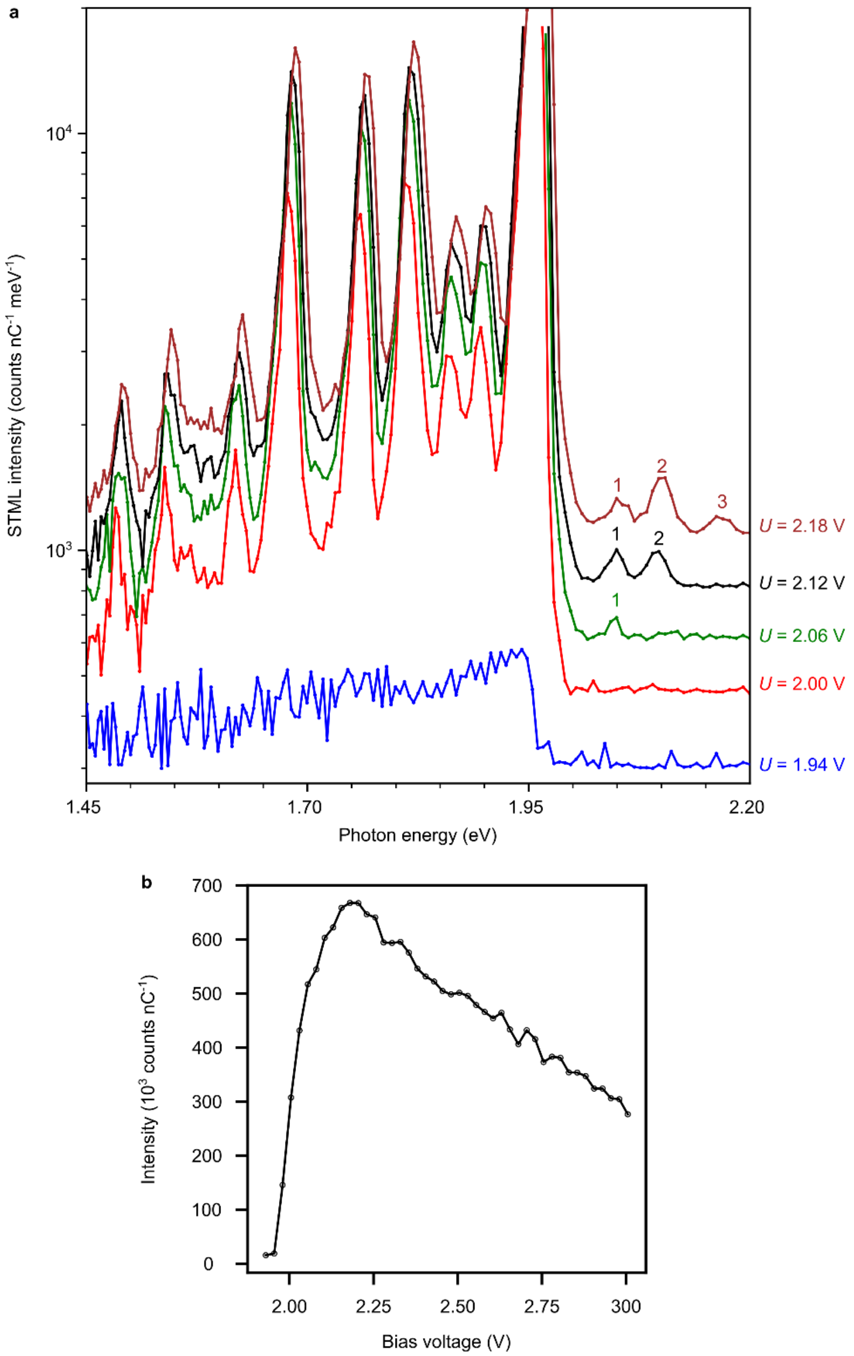

Suppl. Fig. 4 │ STML spectra as a function of applied bias voltage recorded on a single Tol-Tpd-nNDI. a Parameters for recording the spectra are I = 10 pA, t_{exp} = 10 s bias voltage indicated on the right side of each spectrum. **b** Integrated photon counts of Q_n emission line as a function of applied sample bias voltages (I = 0.50 pA, t_{exp} = 10 s).

In Suppl. Fig. 4, the evolution of the STML spectrum as a function of the applied bias voltage is presented. The measurement is performed on a single **Tol-Tpd-nNDI** molecule. At the bias voltage of U = 1.94 V, the Q_n peak just starts to develop (see blue spectrum in Suppl. Fig. 4). Besides, no other peaks are observed. Subsequently, as the energy of the tunnelling electrons

is increased further by increasing the bias voltage, the main emission line Q_n develops together with all the peaks at lower energies (see red spectrum at U = 2.0 V). For the emission of peaks at higher energies (HE-band), the energy of the tunnelling electrons needs to match the energy of the HE peaks as can be seen in the spectra recorded at U = 2.06 V, 2.12 V and 2.18 V. HE peaks are marked with 1, 2 and 3.

Furthermore, we show the bias dependence of the main emission line (Q_n) up-to 3 V. The integrated photon count for the Q_n line first increases followed by a peak and then gradually decreases.

Supplementary Note 5. Current-dependent STML Measurements

Suppl. Fig. 5 shows the integrated photon emission rate of the Q_n line as a function of the tunnelling current. The measurement was performed on a single **Tol-Tpd-nNDI** molecule at a bias voltage of 2.4 V. We observe that the photon count scales linearly with the tunnelling current, suggesting a one-electron process. This supports the argument of the light emission being driven by inelastic energy transfer via plasmons, which has been reported before for single molecules in the STM junction[3–6].

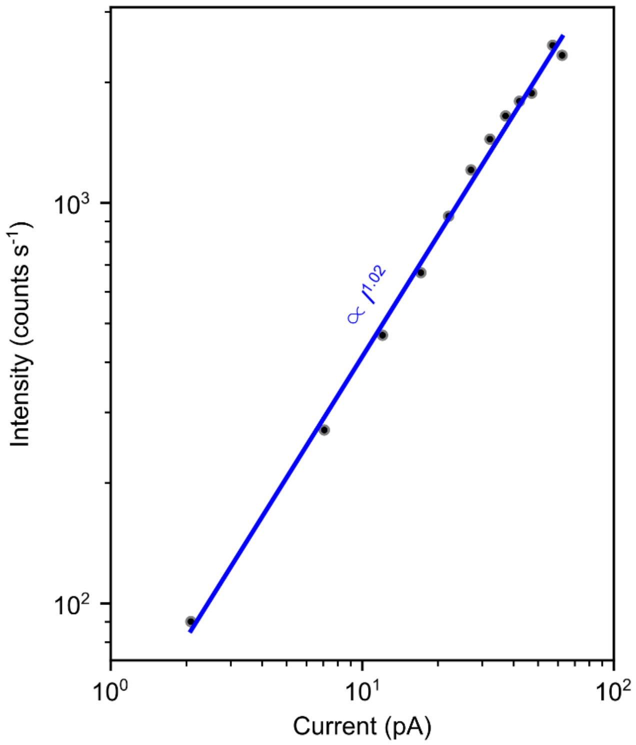

Suppl. Fig. 5 │ Photon count rate integrated over the Q_n line measured as a function of the tunnelling current (U = 2.4 V, t_{exp} = 90 s).

Supplementary Note 6. Time-dependent Density Functional Theory Investigations

For the **Tol-Tpd-nNDI**-Ac and the **Tol-Tpd-sNDI**-Ac molecule, the absorption spectra have been calculated using time-dependent density functional theory. The TMHF functional has been used in conjunction with the def2-TZVP basis set[7]. TMHF is a recently developed local hybrid functional, being derived only from first principles[8]. TMHF has shown excellent performance for excited states and charge-transfer excitations. Therefore, starting from a r^2SCAN optimized geometry[9], the absorption spectra are calculated. Vibrational spectra were accordingly obtained at the r^2SCAN/def2-TZVP level of theory. To correct for the adiabatic excitation shift, we further optimize the first excited state at the TMHF/ def2-TZVP level of theory, re-calculate the excitation energies, and shift the spectra by the obtained energy difference. The local hybrid TMHF was chosen to account for possible charge-transfer excited states. For **Tol-Tpd-nNDI**-Ac, a shift of 0.26 eV is obtained, while for **Tol-Tpd-sNDI**-Ac, a shift of only 0.02 eV of the excited state is observed. The calculated absorption spectra, corrected for the adiabatic energy shift, are shown in Suppl. Fig. 6. Photon maps have been obtained for the excited states shown in Suppl. Fig. 6 using the equation (1)

$$g(\mathbf{r}) = \int \frac{V_{\mathrm{tip}}(\mathbf{r})\rho_{\mathrm{n}}^{\mathrm{ex}}(\mathbf{r}')}{|\mathbf{r}-\mathbf{r}'|} d^3\,\mathbf{r}\, d^3\mathbf{r}' \qquad (1)$$

as described in the references[10,11]. The approximate tip model from the literature[11] was used. V_{tip} refers to the tip potential, while $\rho_{\mathrm{n}}^{\mathrm{ex}}$ refers to the excited state density of the *n*-th excitation. The Coulomb potential of the excited state is evaluated using the semi numerical semiJK algorithm outlined in the reference[12].

Suppl. Table 1 │ List of vibrational modes of Tol-Tpd-nNDI-Ac **ranging from 470-510 cm^{-1}.** All modes centered around 499-501 cm^{-1} are variations of the mode we depicted in the Suppl. Movie 2. The next intense vibration at 494 cm^{-1} is also clearly located at the tripodal scaffold.

Mode Nr.	Freq. (cm^{-1})	Intensity (km mol^{-1})
163	471.4	5.676
164	476.7	1.517
165	477.4	1.591
166	482.2	6.279
167	489.2	0.798
168	489.9	2.771
169	494.2	5.217
170	499.2	16.540
171	500.2	1.339
172	501.4	2.665
173	510.7	0.812

Suppl. Table 2 │ List of vibrational modes of Tol-Tpd-nNDI-Ac **ranging from 1600-1730 cm^{-1}.** These frequencies are all variations of the C=O stretching mode.

Mode Nr.	Freq. (cm^{-1})	Intensity (km mol^{-1})
497	1603.8	31.623
498	1607.5	17.187
499	1608.6	16.926
500	1613.5	7.165
501	1614.0	8.085
502	1616.6	11.677
503	1618.1	0.963
504	1618.8	31.833
505	1655.1	298.056
506	1662.8	131.490
507	1689.2	210.700
508	1696.1	114.087
509	1726.2	156.842
510	1727.8	167.284
511	1730.8	162.164

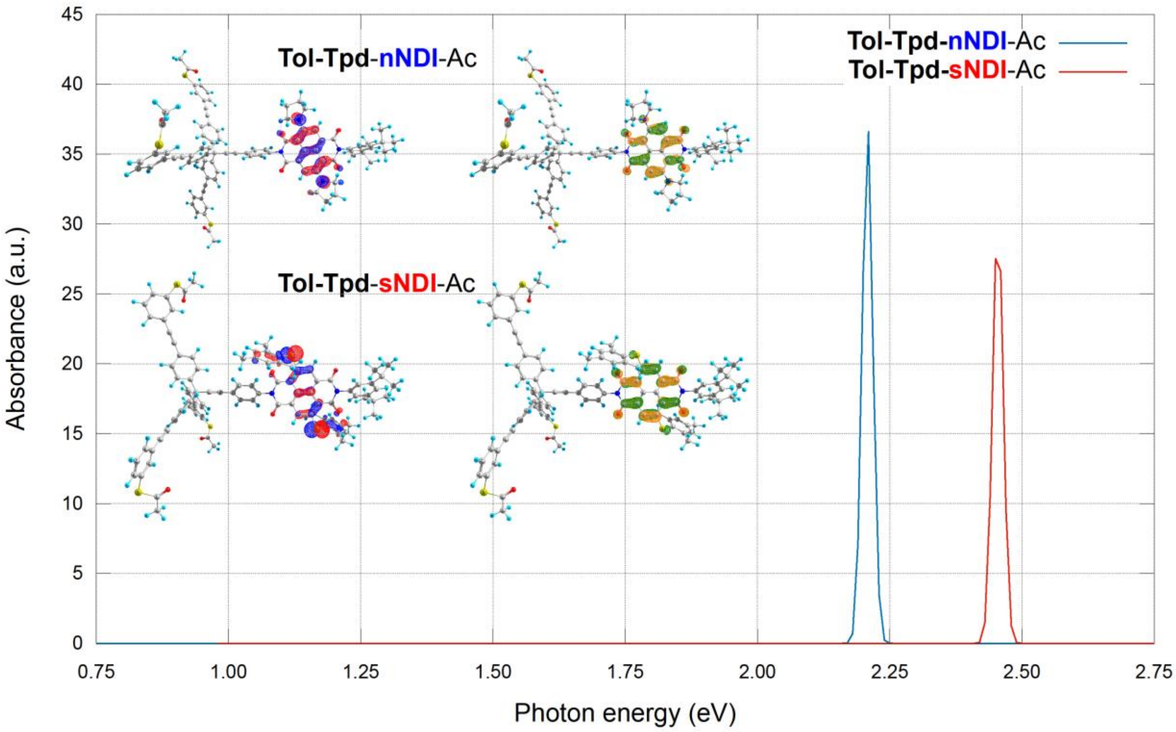

Suppl. Fig. 6 │ Calculated absorption spectra for Tol-Tpd-nNDI-Ac **and Tol-Tpd-sNDI-**Ac**.** Spectra computed using the TD-TMHF/def2-TZVP method. The inlays show the natural transition orbitals for the most prominent peak of each spectrum. Red/blue and green/orange differentiate the phase of the NTOs. The phase is arbitrary; therefore, no sign is assigned. C/H/N/O and S atoms are marked in grey/cyan/dark blue/red and yellow, respectively.

As can be seen in Suppl. Fig. 6, the peaks of **Tol-Tpd-nNDI**-Ac and **Tol-Tpd-sNDI**-Ac are separated by about 0.25 eV, which is in good agreement with the experimentally observed peaks. Overall, a shift of approximately 0.2 eV is observed compared to experiment, again in good agreement with the measured spectra in solution as well as with the peaks observed in the STM measurements. The natural transition orbitals (NTOs) outline that both peaks have a similar origin, being mainly located in the central part of the NDI moiety. Furthermore, a slight contribution of the substituent can be observed. As expected, the NTO analysis also outlined that the tripodal platform does not interfere with the optical excitation, efficiently decoupling the NDI moiety from the surface once it is attached.

The transition dipole moments of **Tol-Tpd-nNDI**-Ac and **Tol-Tpd-sNDI**-Ac are strongly polarized as outlined in Suppl. Table 3.

Suppl. Table 3 │ Transition dipole moments of the first excited state (most prominent peak). All values in atomic units.

Molecule	D_x^{trans}	D_y^{trans}	D_z^{trans}
**Tol-Tpd-nNDI**-Ac	-1.567	-0.021	1.219
**Tol-Tpd-sNDI**-Ac	1.654	0.568	0.413

The transition dipole moments in Suppl. Table 3 outline in which direction light will be emitted (or absorbed) most efficiently. By inspecting Suppl. Fig. 7, which depicts the orientation of **Tol-Tpd-nNDI**-Ac and **Tol-Tpd-sNDI**-Ac molecules, strong bands can be expected for both molecules into the *x*-direction, parallel to the NDI system and perpendicular to the main *z*-axis. Also, for **Tol-Tpd-nNDI**-Ac, a strong peak in *z*-direction can be observed, as required for the outgoing observed light in the STML experiment carried out in this work. This peak is significant for **Tol-Tpd-sNDI**-Ac, too, but reduced to roughly one third of the intensity. As the emission probability is proportional to the square of the transition dipole moment elements, this translates to a roughly 9 times more efficient emission of **Tol-Tpd-nNDI**-Ac when compared to **Tol-Tpd-sNDI**-Ac. Contrary, no intensity can be observed for **Tol-Tpd-nNDI**-Ac in *y*-direction, perpendicular to the NDI system. **Tol-Tpd-sNDI**-Ac shows a more isotropic distribution of the transition dipole moment in *y*- and *z*-direction, probably due to the larger spatial extend of the core NDI substituent, which also extends further away from the plane spanned by the NDI core.

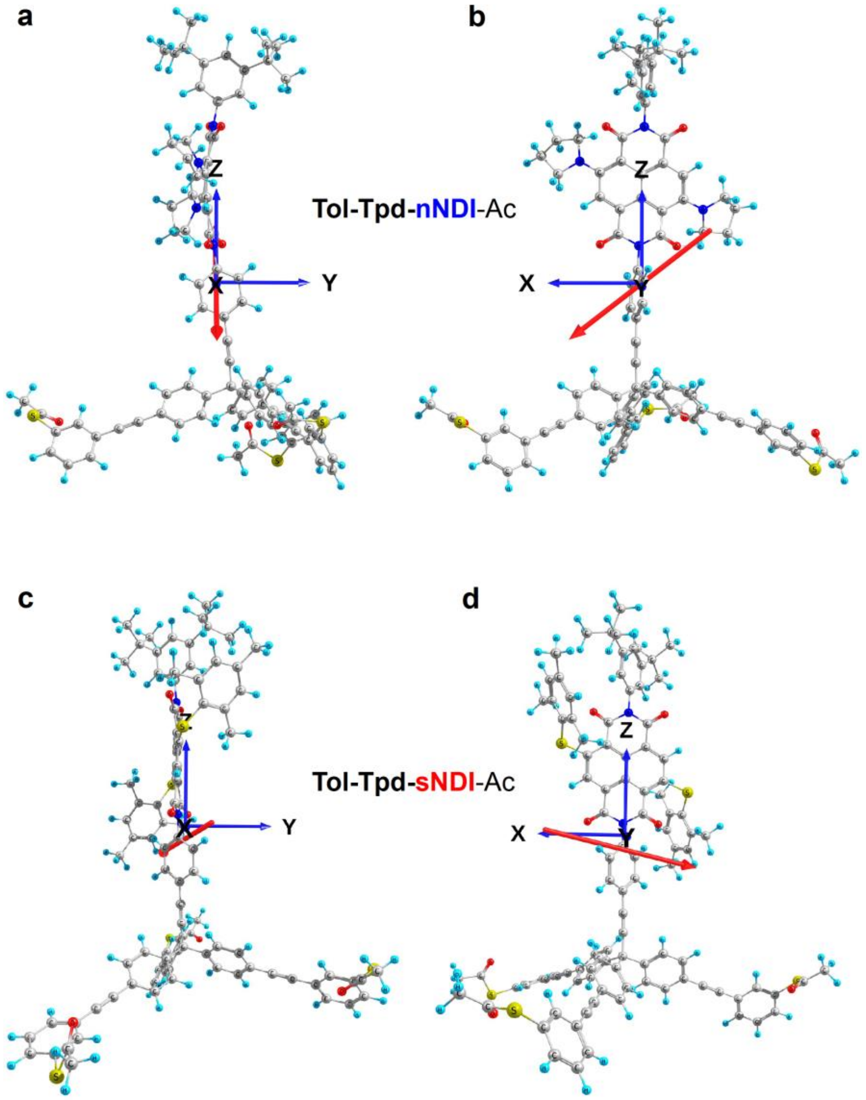

Suppl. Fig. 7 │ Orientation of Tol-Tpd-nNDI-Ac **and Tol-Tpd-sNDI**-Ac **obtained from DFT optimization in vacuum**. Drawn as red arrows are the calculated transition dipole moments of the respective first excited state at the TMHF/def2-TZVP level of theory. Inset **a** and **c** show the side of the NDI core, being aligned in the *xz*-plane, while **b** and **d** show the top view on the NDI core, with the *y*-axis pointing towards the viewer. C/H/N/O and S atoms are marked in grey/cyan/dark blue/red and yellow, respectively.

From the difference in transition dipole moment alone, it can however not be explained why the STML efficiency of **Tol-Tpd-nNDI**-Ac is three orders of magnitude higher than that of **Tol-Tpd-sNDI**-Ac, as the latter can only account for one order of magnitude. The reason for the remaining deviations is that for **Tol-Tpd-sNDI**-Ac an initially higher lying excited state can fall below the S_1 state depicted in Suppl. Fig. 6. Optimizing the geometry of the first excited state of **Tol-Tpd-sNDI**-Ac leads to an excited state with considerably lower energy, being located at around 1.45 eV. This state is identified as a charge-transfer state, where charge is transferred from the 2,4,6-trimethylphenylsulfanyl moiety to the central NDI core. The first excited state at the ground state geometry can be identified as the S_2 state on the full hypersurface. Contrary, for **Tol-Tpd-nNDI**-Ac the S_1 state at the ground state geometry is also the S_1 state of the full

hypersurface. Therefore, for **Tol-Tpd-sNDI**-Ac additional relaxation pathways exist, further lowering the efficiency of light emission which is experimentally observed at 2.25 eV, in full agreement with the experimental observation of about three orders of magnitude lower efficiency.

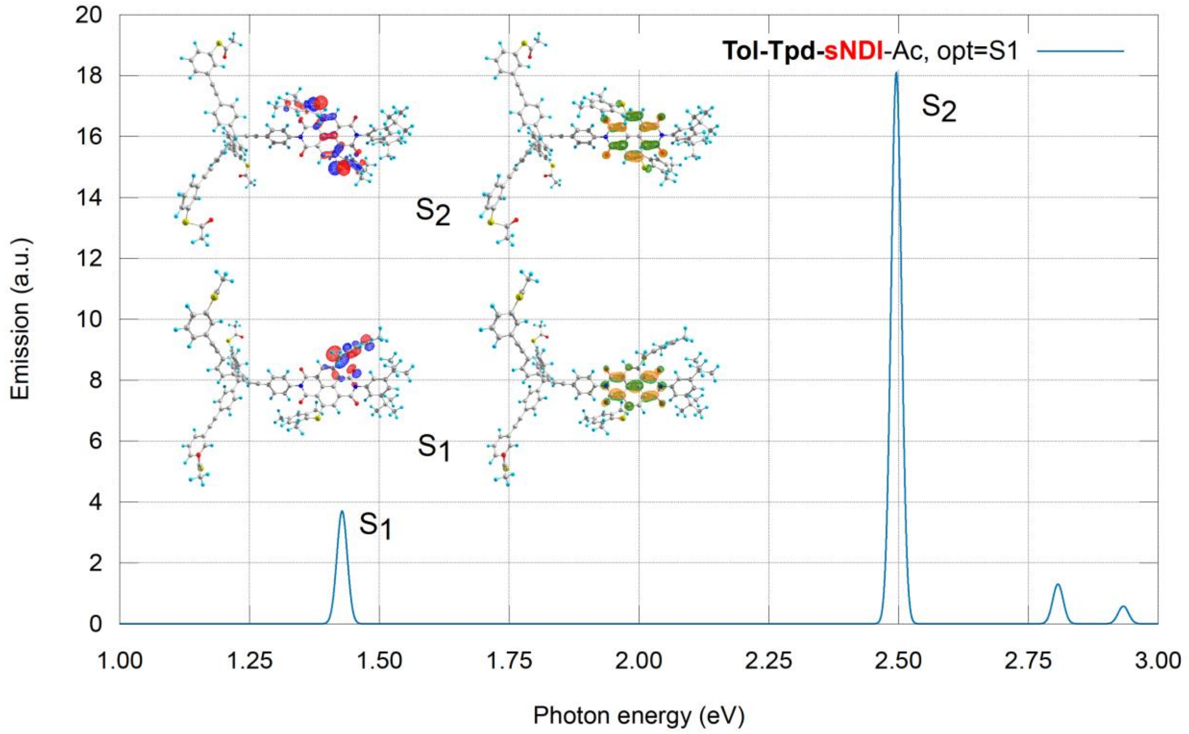

Suppl. Fig. 8 │ Calculated emission spectrum for Tol-Tpd-sNDI-Ac. Spectrum computed using the TD-TMHF/def2-TZVP method. The inlays show the natural transition orbitals for the S_1 and S_2 states. Red/blue and green/orange differentiate the phase of the NTOs. The phase is arbitrary; therefore, no sign is assigned. C/H/N/O and S atoms are marked in grey/cyan/dark blue/red and yellow, respectively.

Supplementary Note 7. Spectrophotometric Studies of Tol-Tpd-sNDI-Ac and Tol-Tpd-nNDI-Ac

UV-Vis absorption and fluorescence spectra were recorded in dichloromethane with a concentration of 25 µM in a 1 cm quartz cell at ambient temperature and are shown in Suppl. Fig. 9. The molecule **Tol-Tpd-sNDI**-Ac was excited at 370 nm and both excitation and emission resolution were set to 20 nm, while the molecule **Tol-Tpd-nNDI**-Ac was excited at 366 nm and both excitation and emission resolution were set to 10 nm.

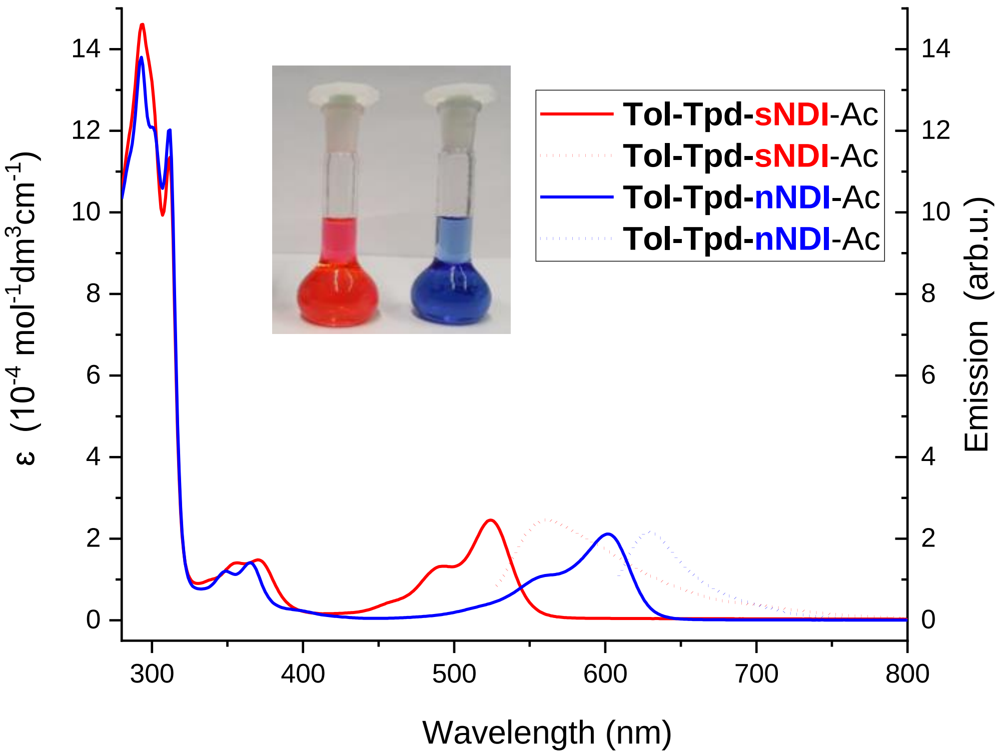

Suppl. Fig. 9 │ UV-Vis absorption spectra (solid line) of Tol-Tpd-sNDI-Ac **(red) and Tol-Tpd-nNDI-**Ac **(blue) molecules and their normalized emission spectra (dotted lines).** Recorded in dichloromethane with a concentration of 25 µM at ambient temperature. A photograph of **Tol-Tpd-sNDI-**Ac (left) and **Tol-Tpd-nNDI**-Ac (right) solutions in dichloromethane is shown in the inset.

Supplementary Note 8. NMR Spectra of all new Compounds

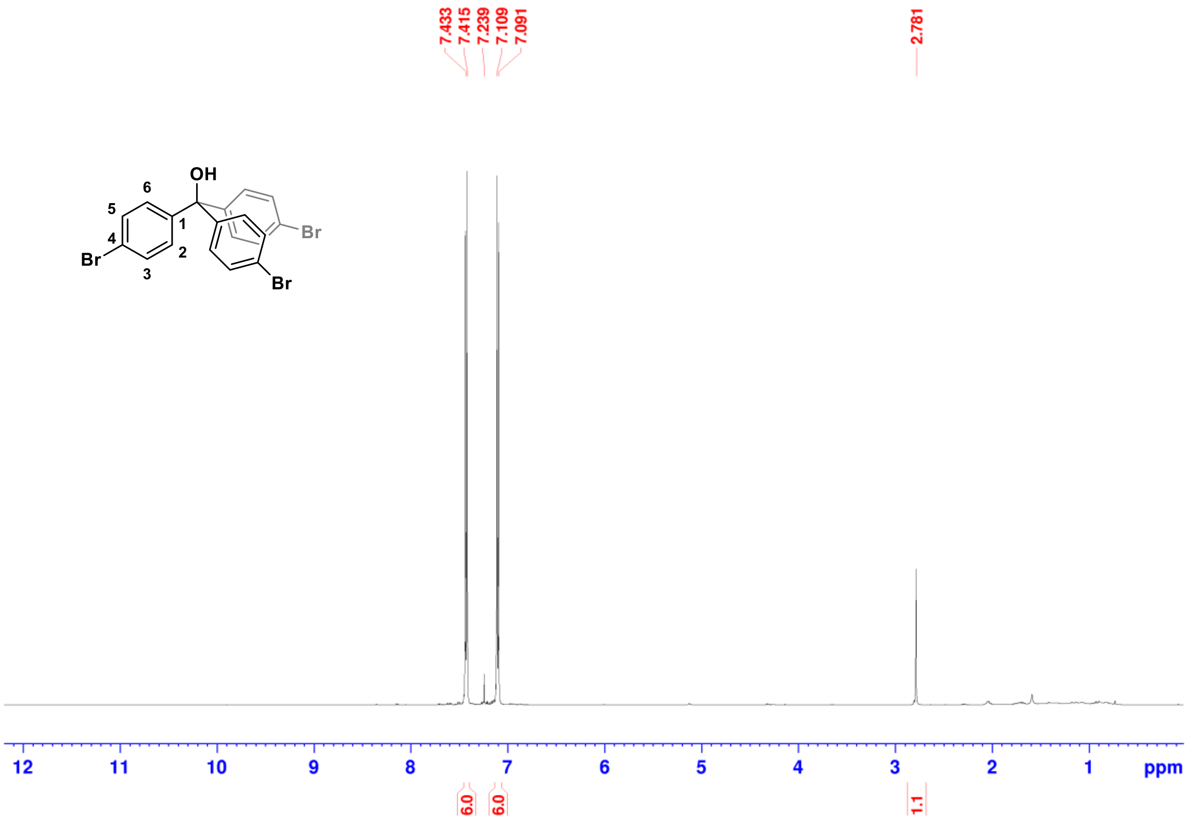

Suppl. Fig. 10 │ ^{1}H NMR (500 MHz, $CDCl_3$) of compound **1**.

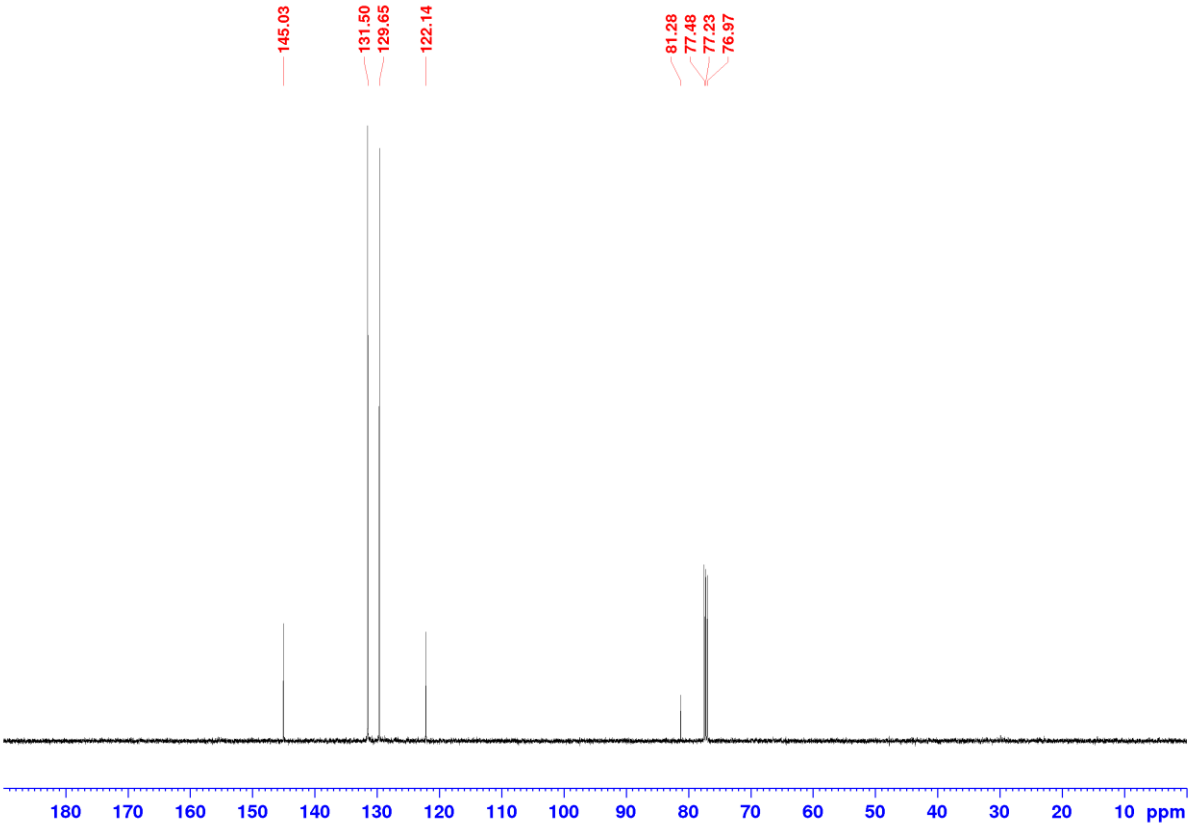

Suppl. Fig. 11 │ ^{13}C NMR (126 MHz, $CDCl_3$) of compound **1**.

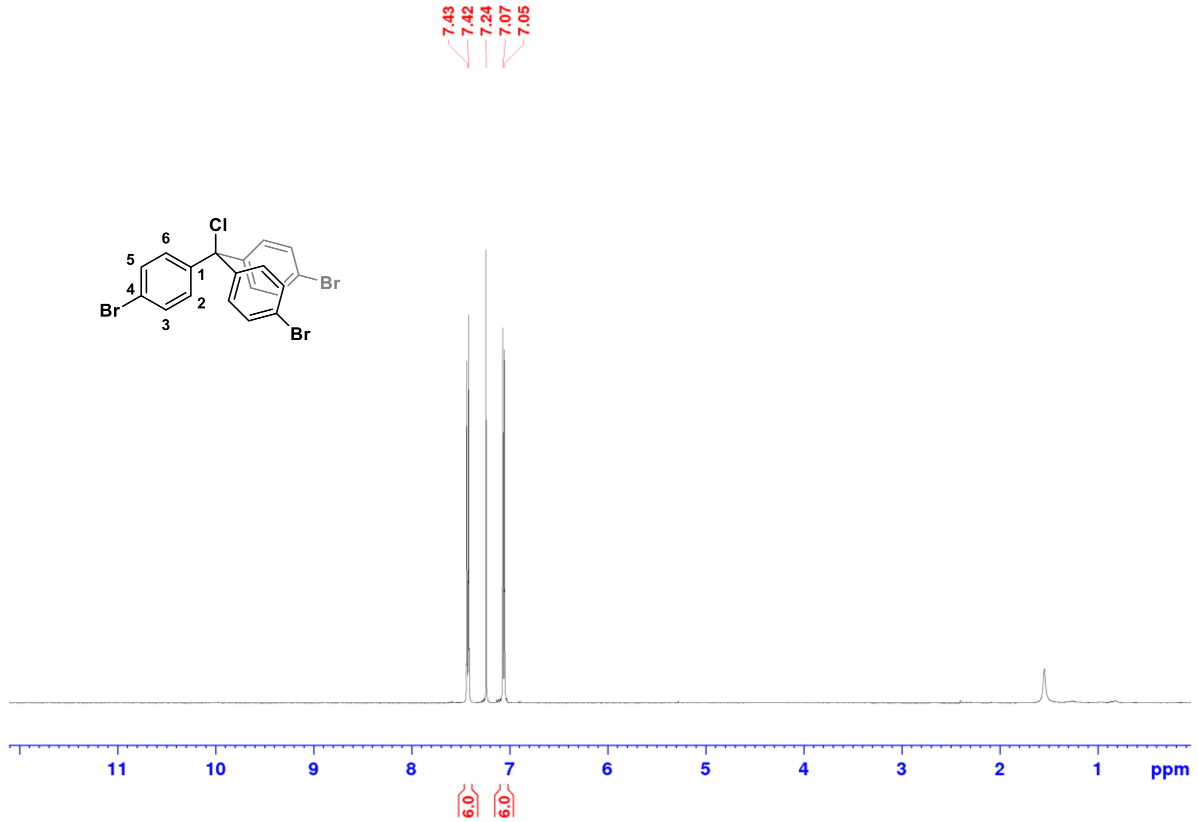

Suppl. Fig. 12 │ ^{1}H NMR (500 MHz, $CDCl_3$) of compound **2**.

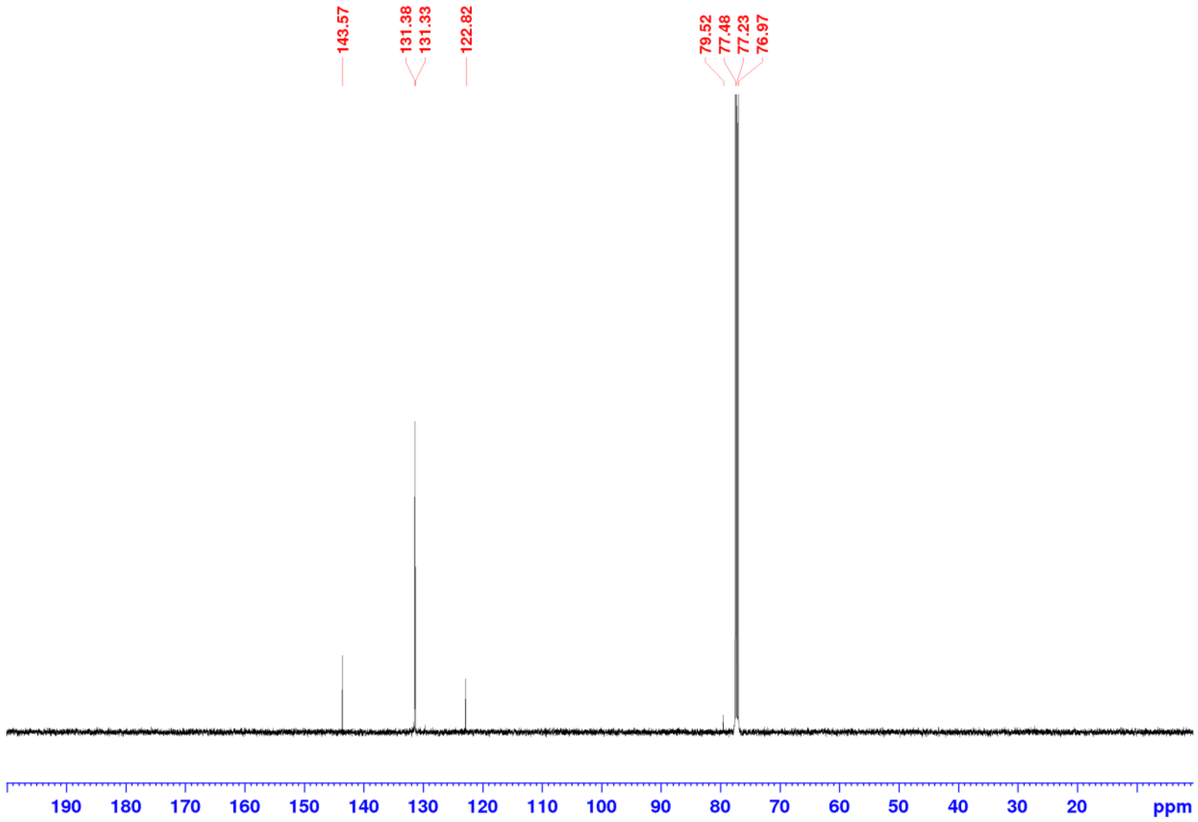

Suppl. Fig. 13 │ ^{13}C NMR (126 MHz, $CDCl_3$) of compound **2**.

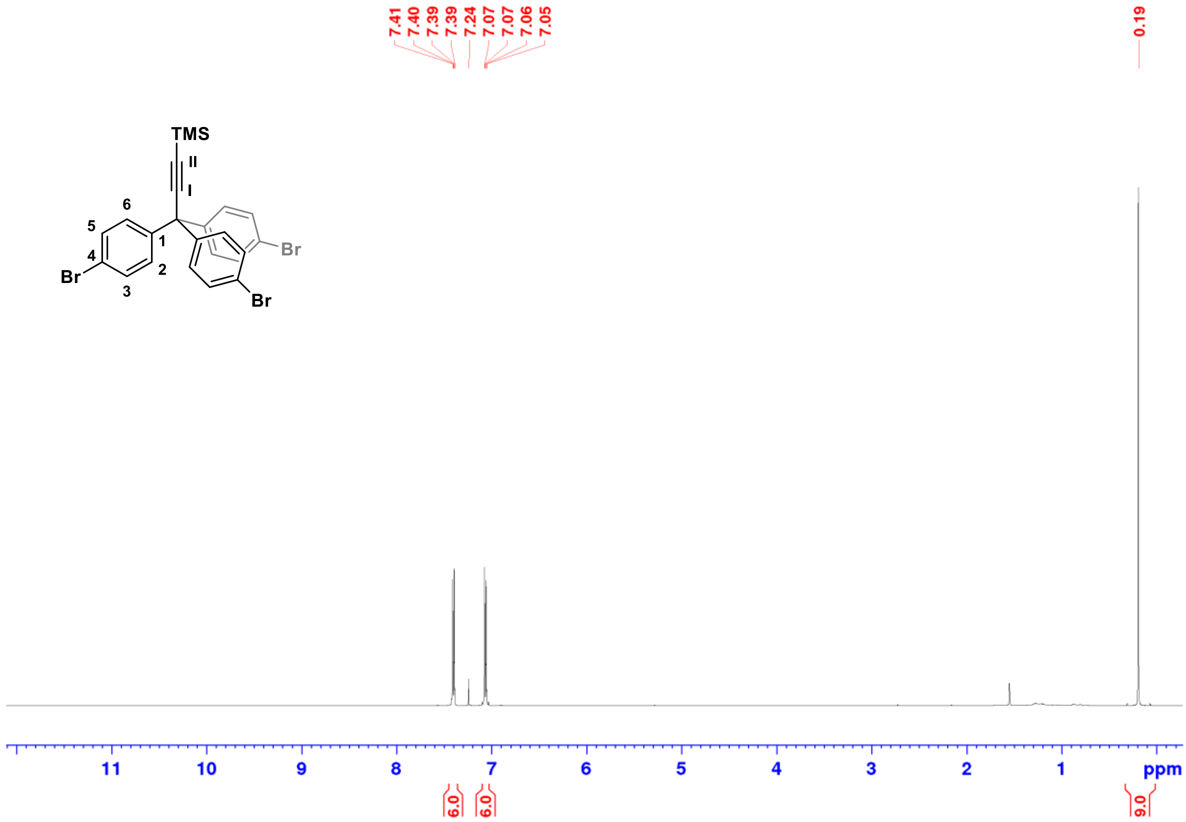

Suppl. Fig. 14 │ ^{1}H NMR (500 MHz, $CDCl_3$) of compound **3**.

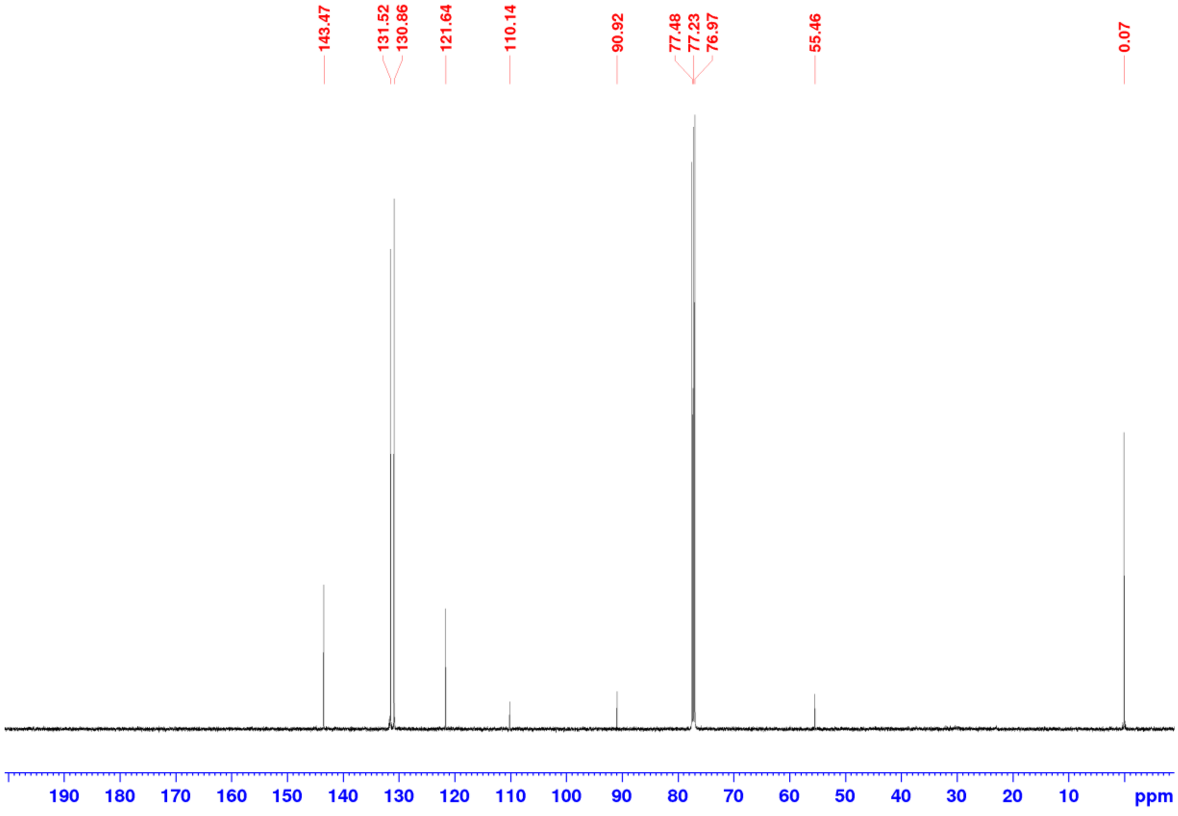

Suppl. Fig. 15 │ ^{13}C NMR (126 MHz, $CDCl_3$) of compound **3**.

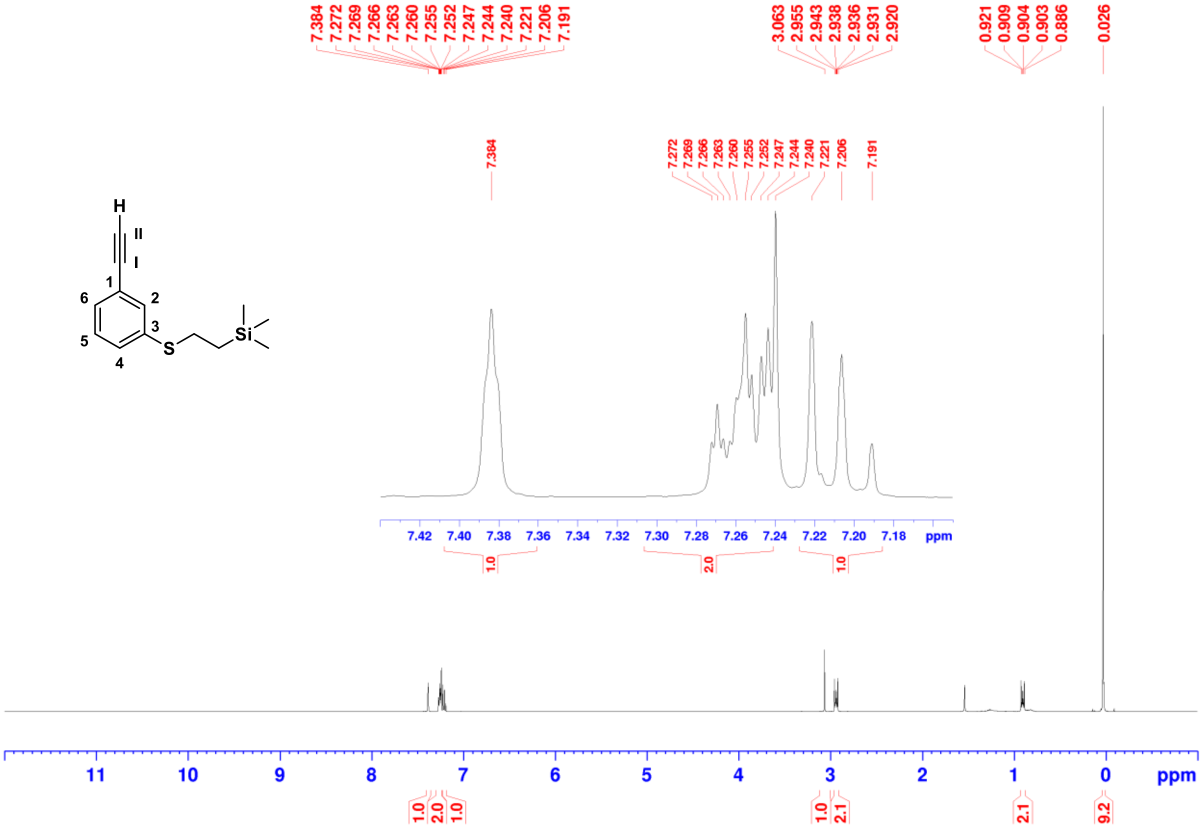

Suppl. Fig. 16 │ ^{1}H NMR (500 MHz, $CDCl_3$) of compound **5**.

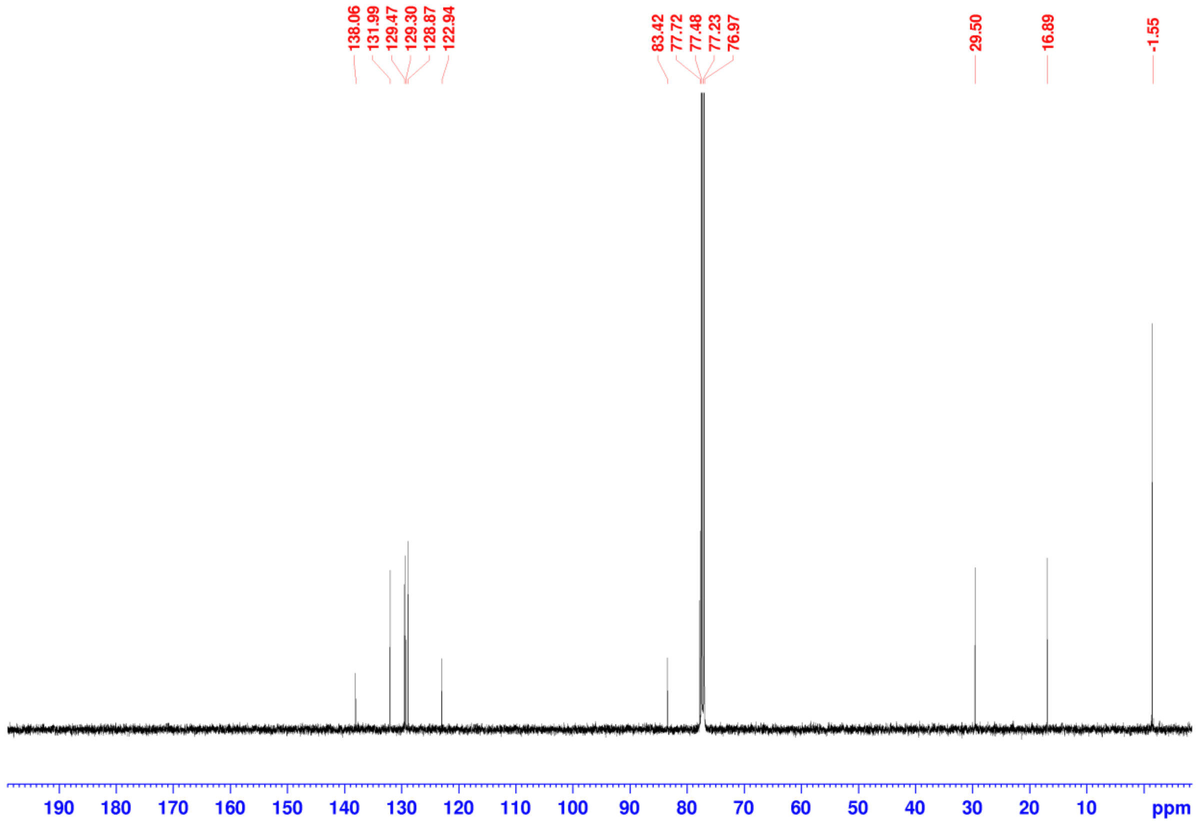

Suppl. Fig. 17 │ ^{13}C NMR (126 MHz, $CDCl_3$) of compound **5**.

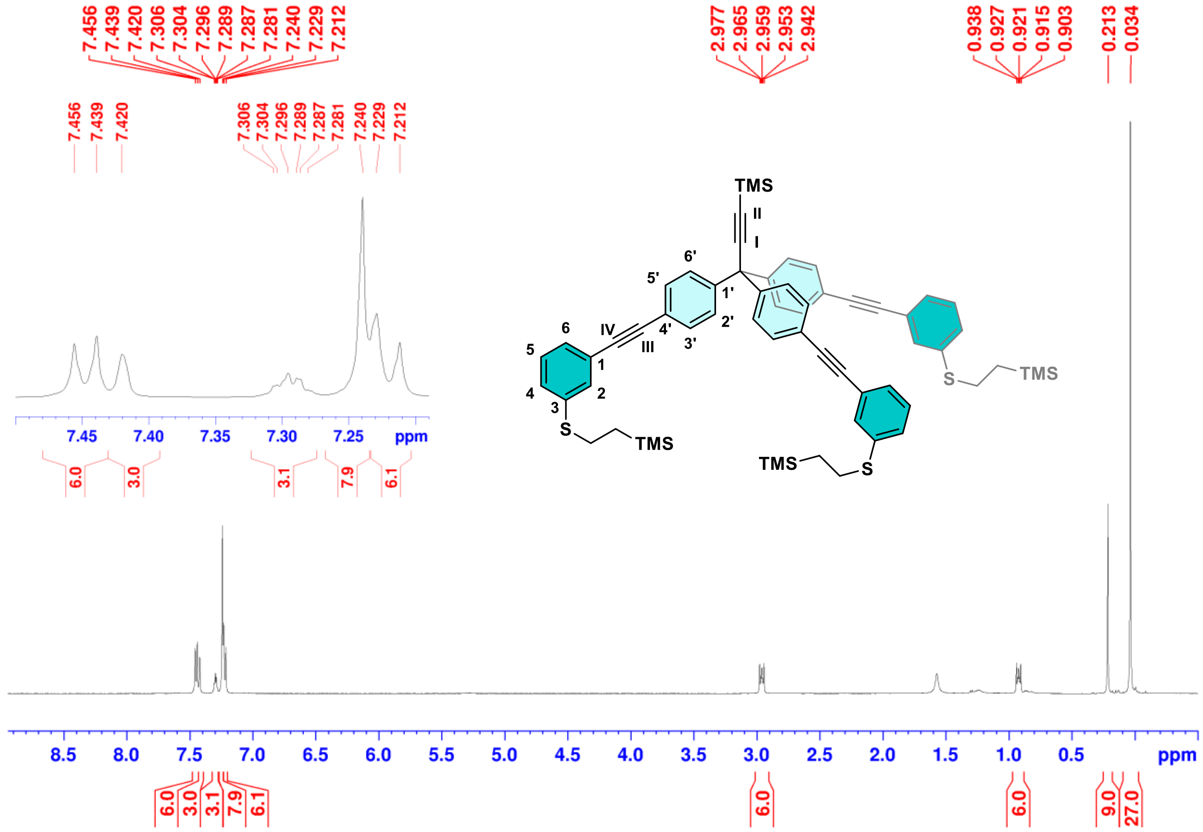

Suppl. Fig. 18 │ ^{1}H NMR (500 MHz, $CDCl_3$) of compound **Tol-Tpd**-TMS.

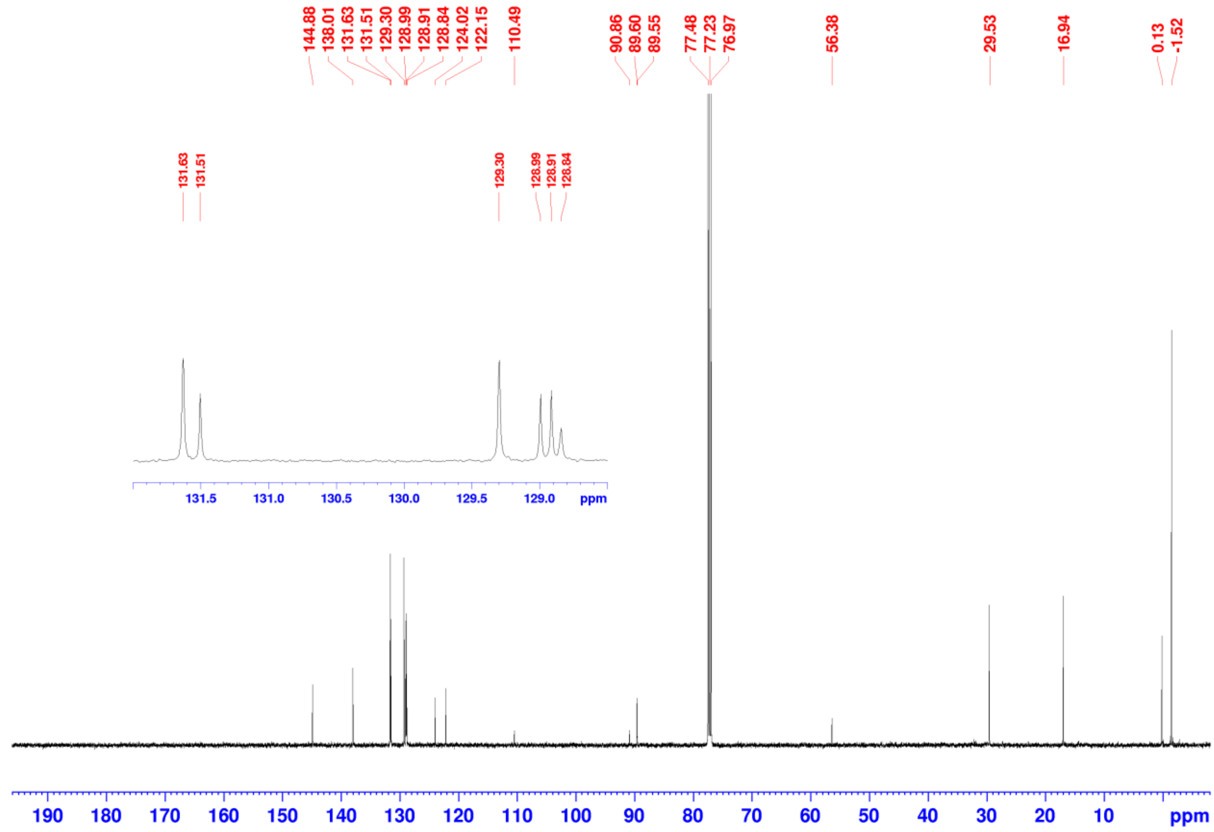

Suppl. Fig. 19 │ ^{13}C NMR (126 MHz, $CDCl_3$) of compound **Tol-Tpd**-TMS.

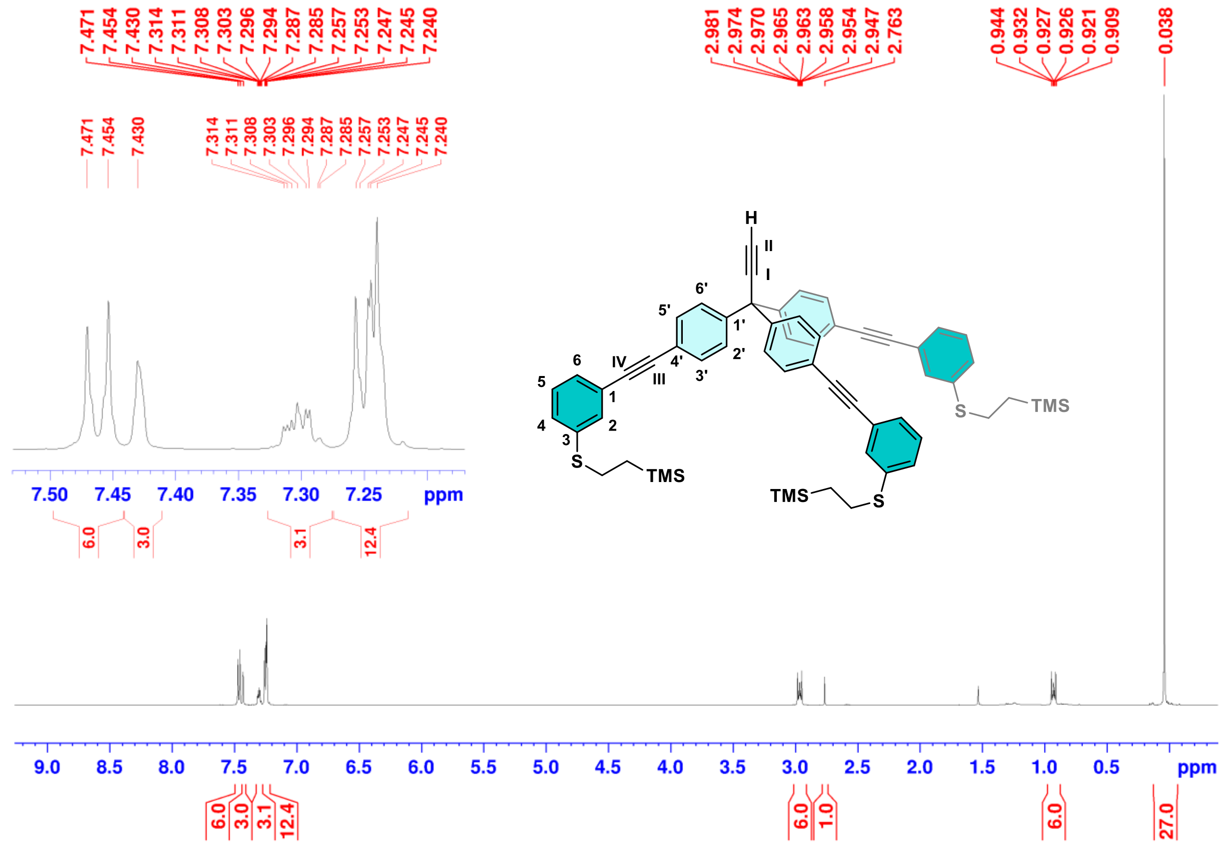

Suppl. Fig. 20 │ ^{1}H NMR (500 MHz, $CDCl_3$) of compound **Tol-Tpd**-H.

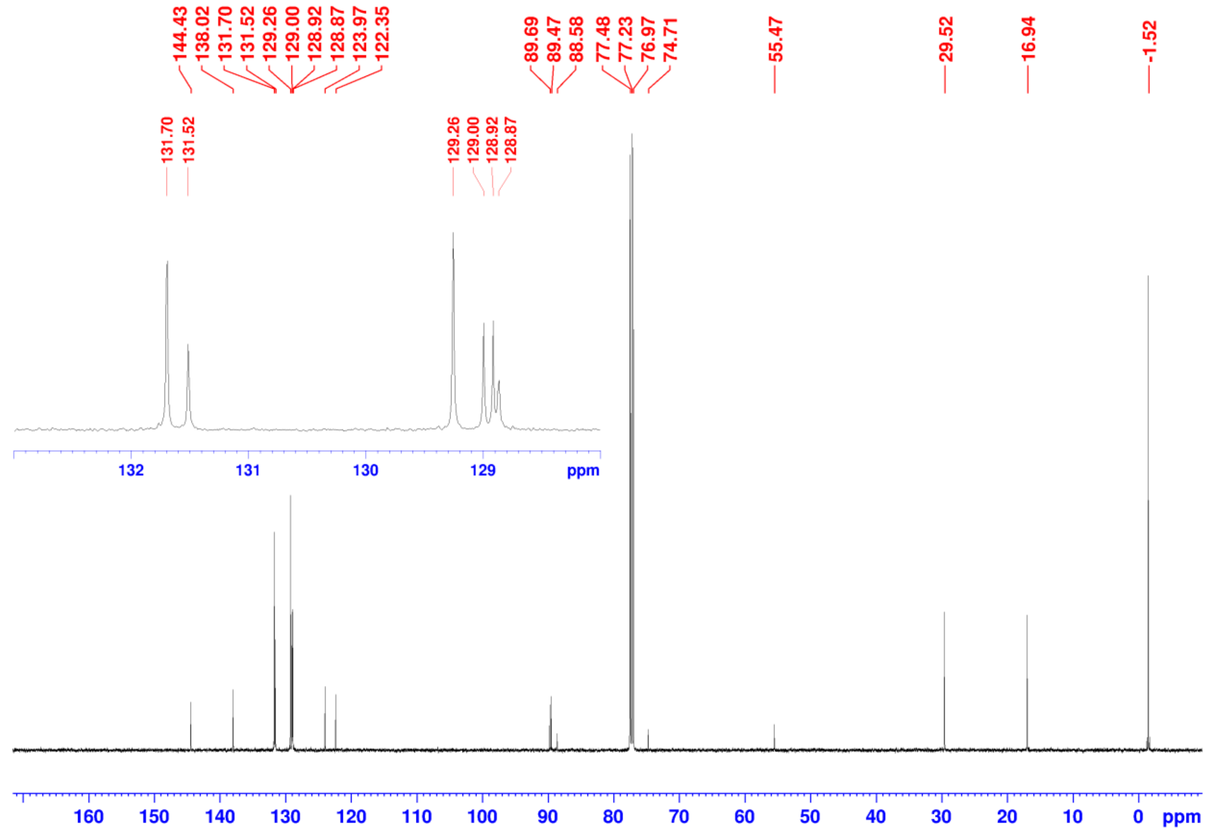

Suppl. Fig. 21 │ ^{13}C NMR (126 MHz, $CDCl_3$) of compound **Tol-Tpd**-H.

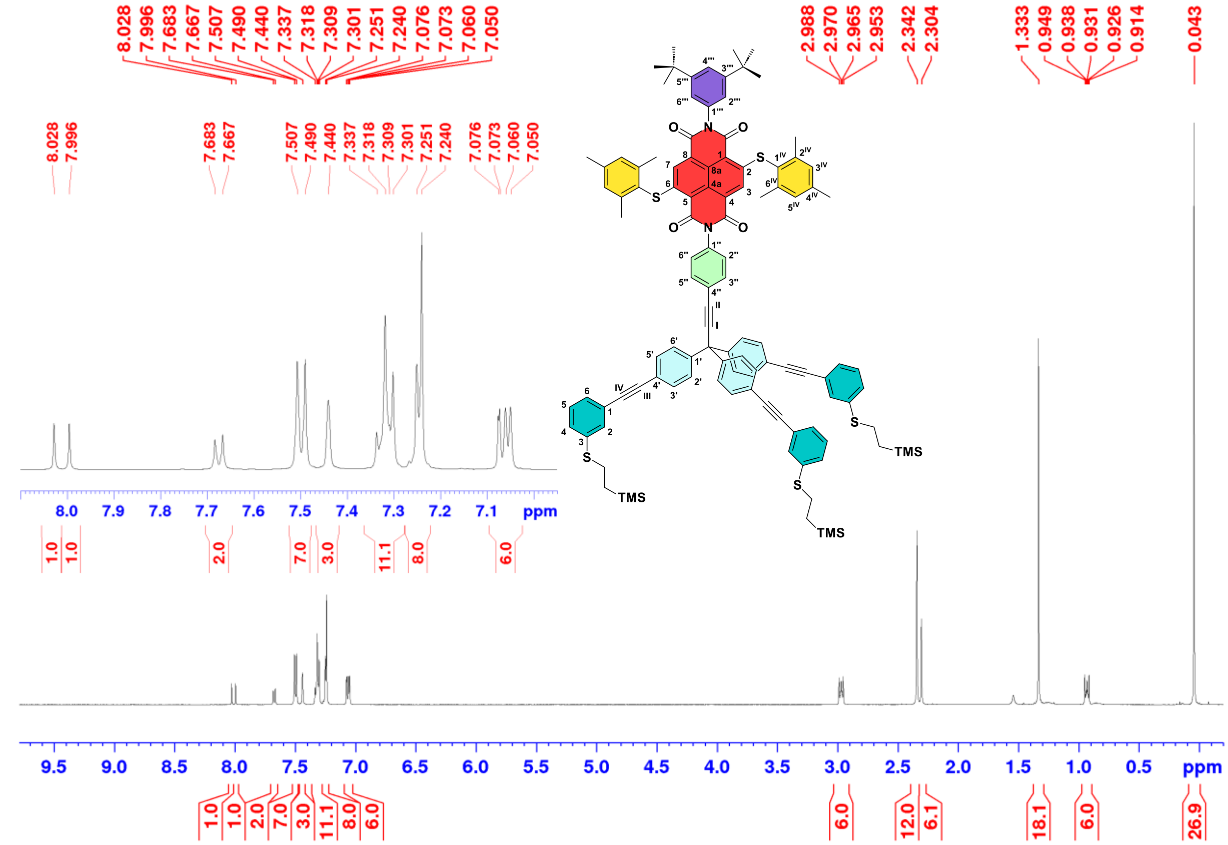

Suppl. Fig. 22 │ ^{1}H NMR (500 MHz, $CDCl_3$) of compound **Tol-Tpd-sNDI**-TMSE.

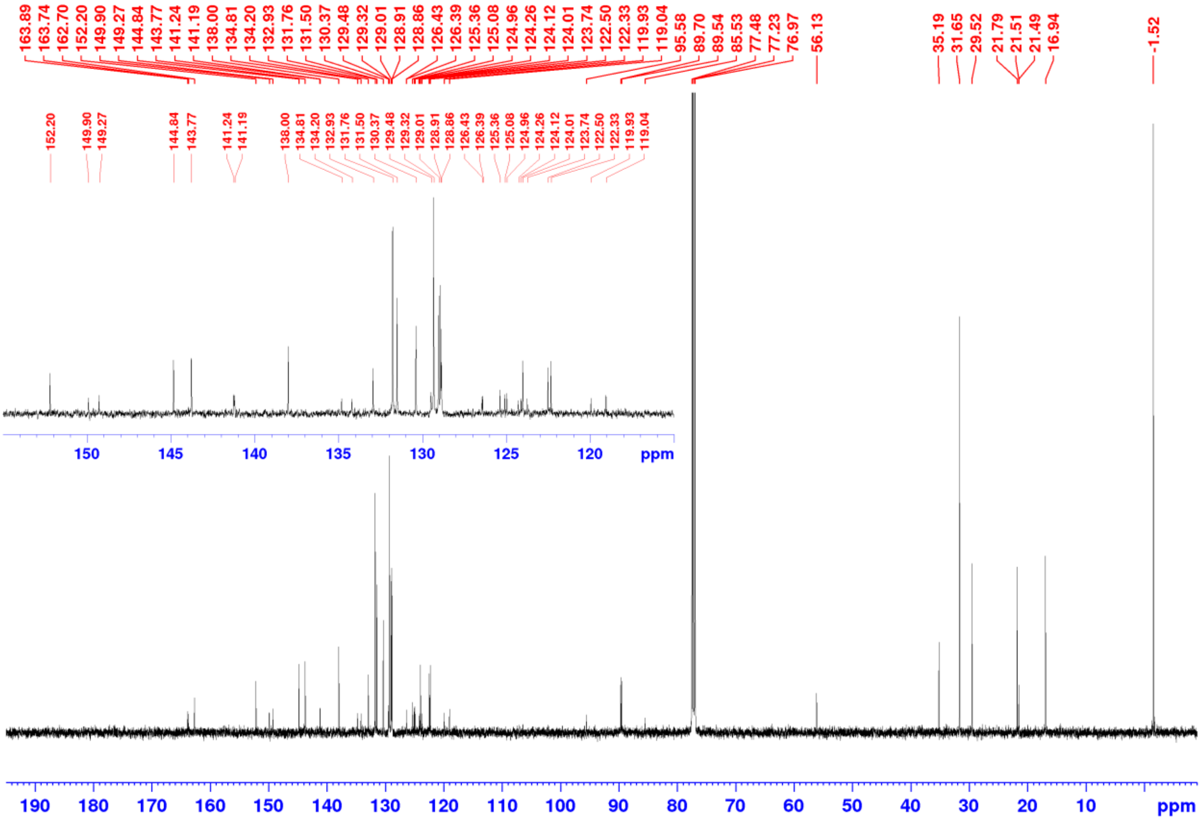

Suppl. Fig. 23 │ ^{13}C NMR (126 MHz, $CDCl_3$) of compound **Tol-Tpd-sNDI**-TMSE.

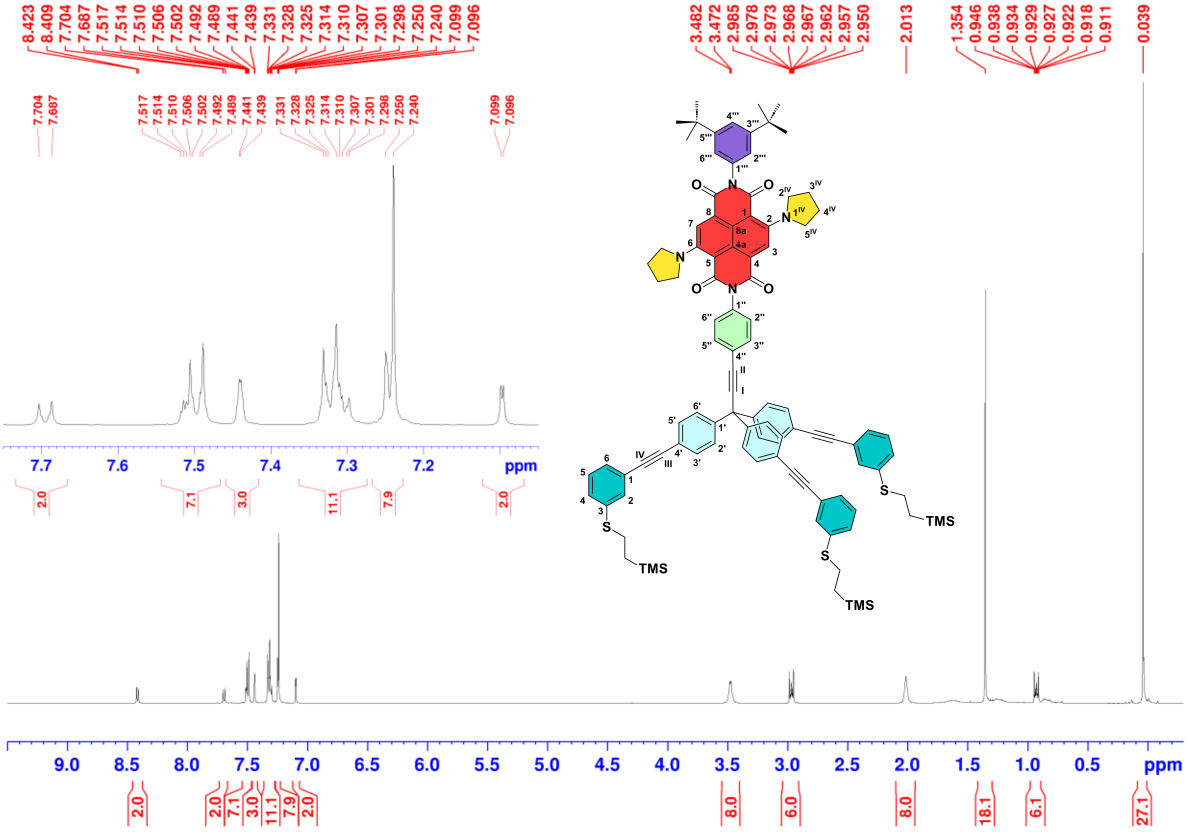

Suppl. Fig. 24 │ ^{1}H NMR (500 MHz, $CDCl_3$) of compound **Tol-Tpd-nNDI**-TMSE.

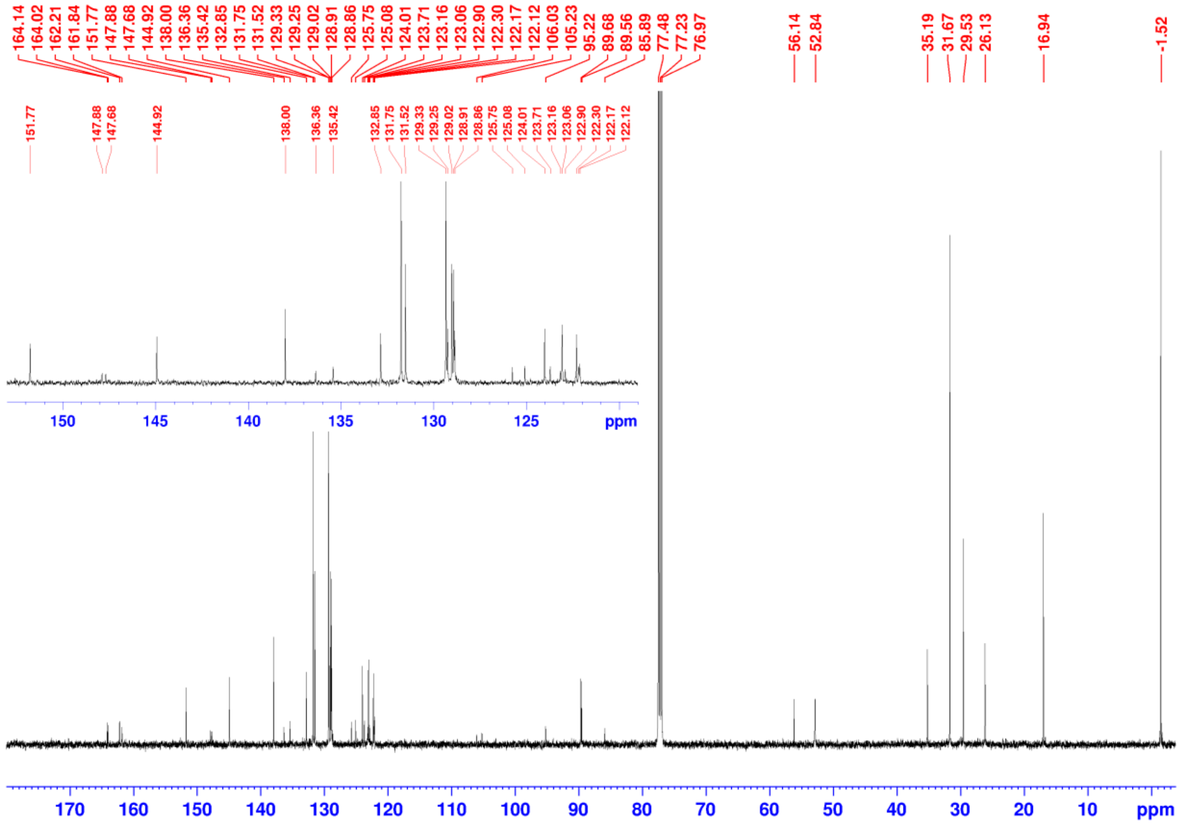

Suppl. Fig. 25 │ ^{13}C NMR (126 MHz, $CDCl_3$) of compound **Tol-Tpd-nNDI**-TMSE.

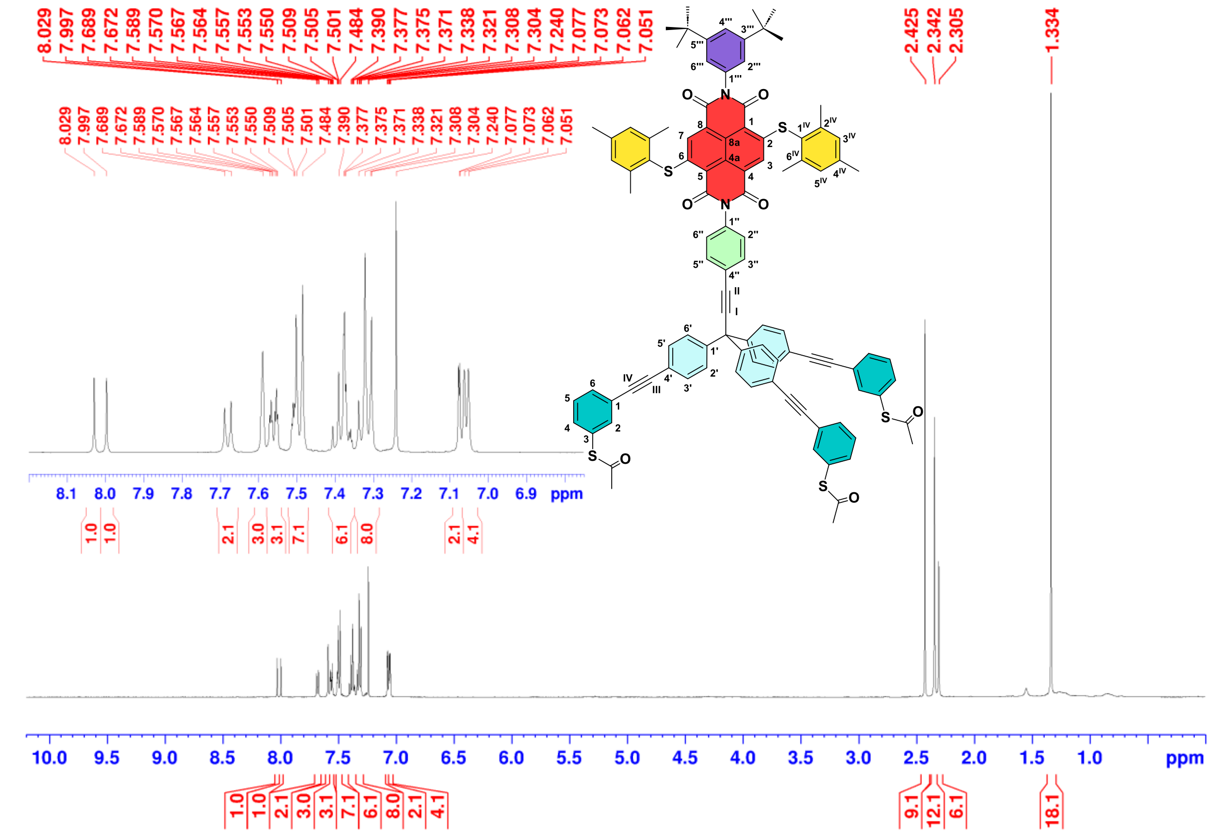

Suppl. Fig. 26 │ ^1H NMR (500 MHz, $CDCl_3$) of compound **Tol-Tpd-sNDI**-Ac.

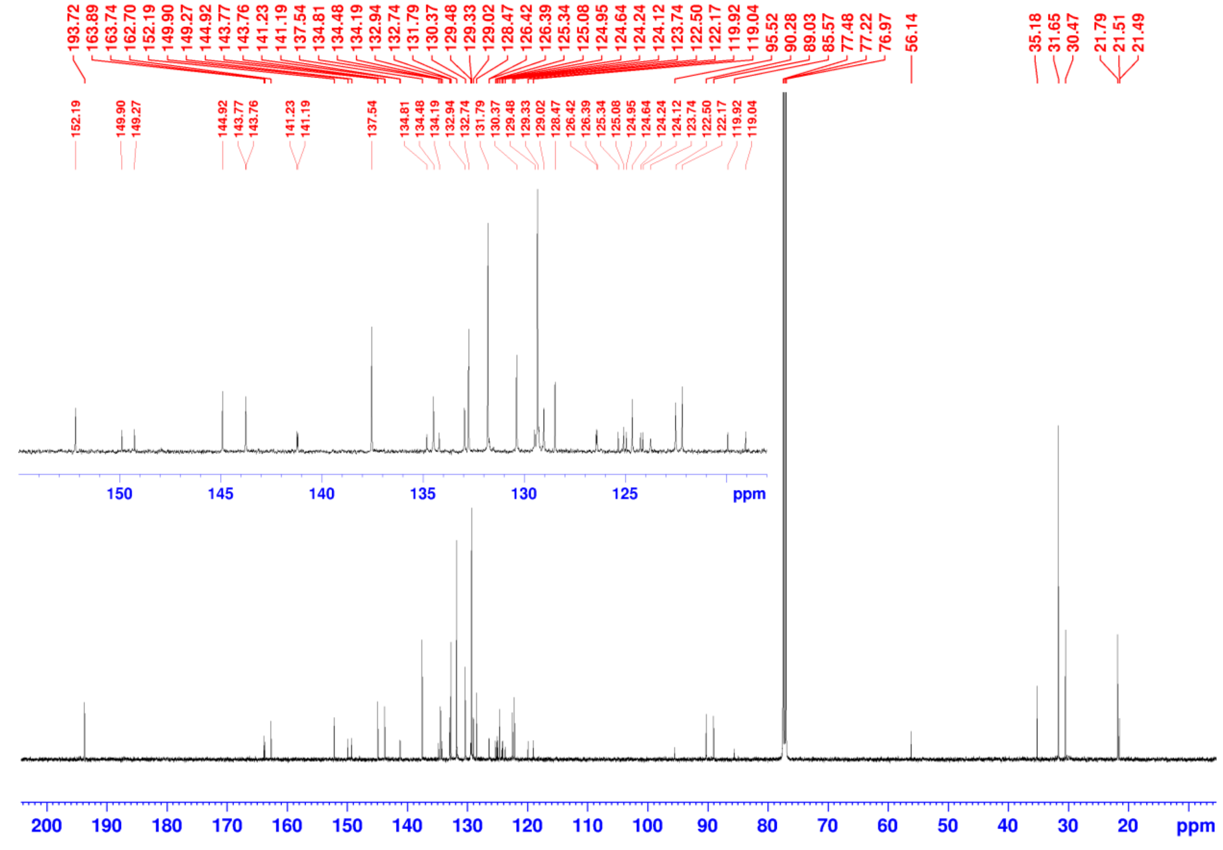

Suppl. Fig. 27 │ ^{13}C NMR (126 MHz, $CDCl_3$) of compound **Tol-Tpd-sNDI**-Ac.

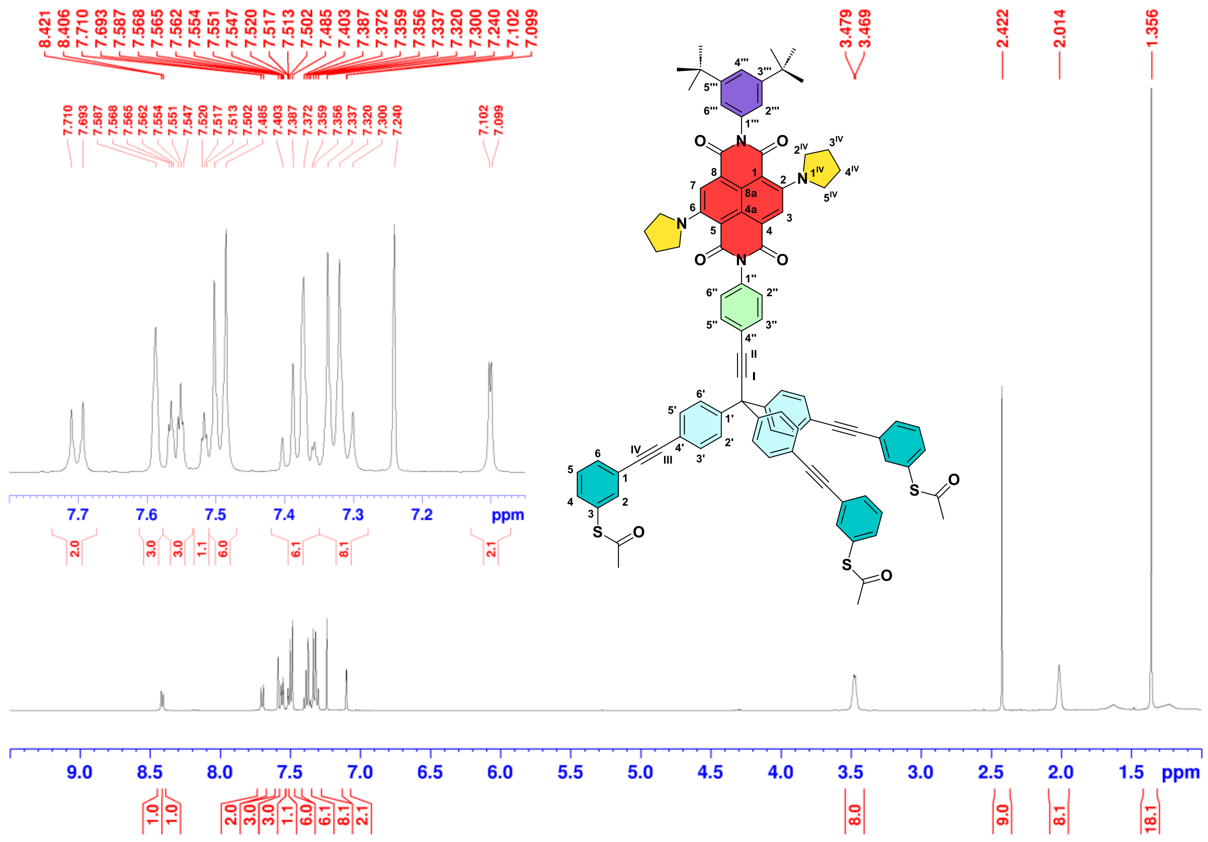

Suppl. Fig. 28 │ ^{1}H NMR (500 MHz, $CDCl_3$) of compound **Tol-Tpd-nNDI**-Ac.

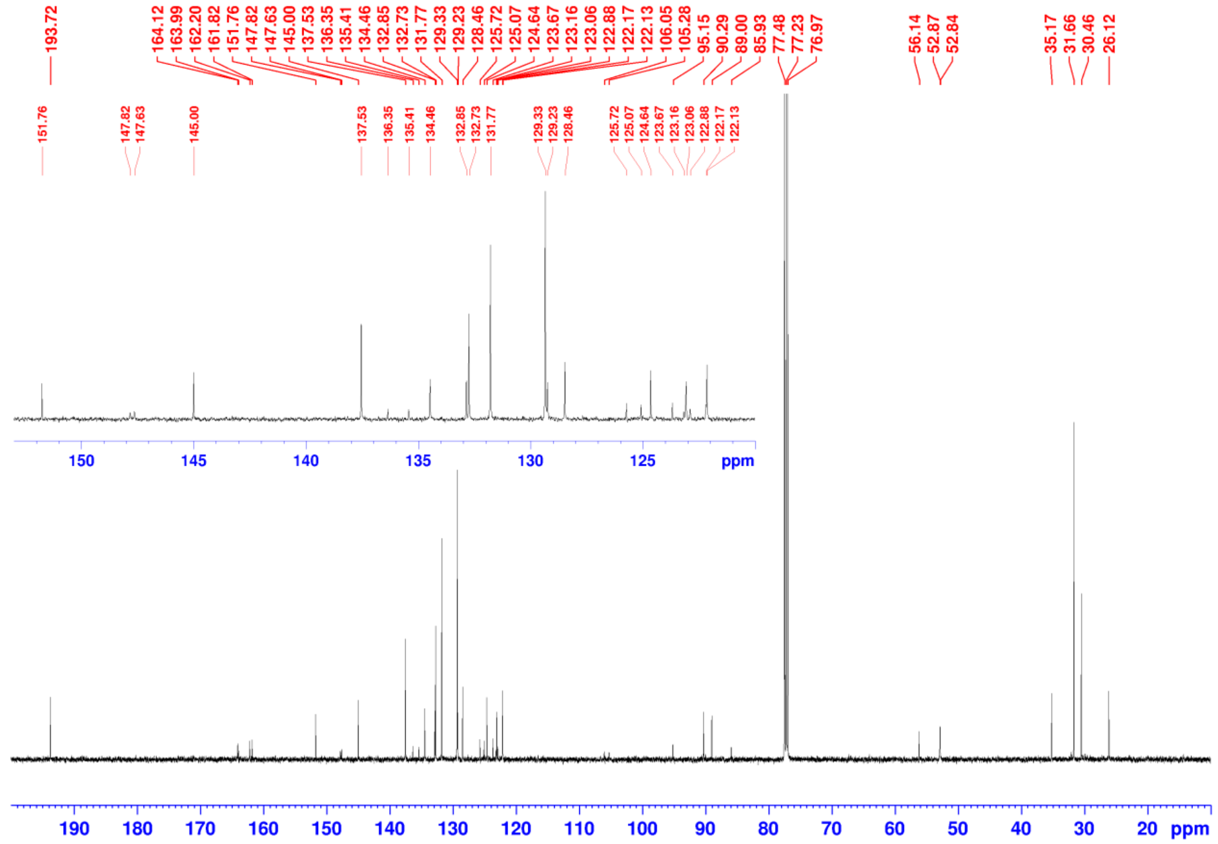

Suppl. Fig. 29 │ ^{13}C NMR (126 MHz, $CDCl_3$) of compound **Tol-Tpd-nNDI**-Ac.

Supplementary References

1. Bunck, D. N. & Dichtel, W. R. Internal Functionalization of Three-Dimensional Covalent Organic Frameworks. *Angew. Chem. Int. Ed.* **51**, 1885–1889 (2012).
2. Homberg, J. *et al.* Six state molecular revolver mounted on a rigid platform. *Nanoscale* **11**, 9015–9022 (2019).
3. Chong, M. C. *et al.* Narrow-Line Single-Molecule Transducer between Electronic Circuits and Surface Plasmons. *Phys. Rev. Lett.* **116**, 036802 (2016).
4. Doppagne, B. *et al.* Electrofluorochromism at the single-molecule level. *Science* **361**, 251–255 (2018).
5. Rai, V. *et al.* Boosting Light Emission from Single Hydrogen Phthalocyanine Molecules by Charging. *Nano Lett.* **20**, 7600–7605 (2020).
6. Dong, Z. C. *et al.* Generation of molecular hot electroluminescence by resonant nanocavity plasmons. *Nat. Photon.* **4**, 50–54 (2010).
7. Weigend, F. & Ahlrichs, R. Balanced basis sets of split valence, triple zeta valence and quadruple zeta valence quality for H to Rn: Design and assessment of accuracy. *Phys. Chem. Chem. Phys.* **7**, 3297–3305 (2005).
8. Holzer, C. & Franzke, Y. J. A local hybrid exchange functional approximation from first principles. *J. Chem. Phys.* **157**, 034108 (2022).
9. Furness, J. W., Kaplan, A. D., Ning, J., Perdew, J. P. & Sun, J. Accurate and Numerically Efficient r2SCAN Meta-Generalized Gradient Approximation. *J. Phys. Chem. Lett.* **11**, 8208–8215 (2020).
10. Neuman, T., Esteban, R., Casanova, D., García-Vidal, F. J. & Aizpurua, J. Coupling of Molecular Emitters and Plasmonic Cavities beyond the Point-Dipole Approximation. *Nano Lett.* **18**, 2358–2364 (2018).
11. Doležal, J. *et al.* Real Space Visualization of Entangled Excitonic States in Charged Molecular Assemblies. *ACS Nano* **16**, 1082–1088 (2022).

12. Holzer, C. An improved seminumerical Coulomb and exchange algorithm for properties and excited states in modern density functional theory. *J. Chem. Phys.* **153**, 184115 (2020).
