# Supplementary material for: Hot luminescence from single-molecule chromophores electrically and mechanically self-decoupled by tripodal scaffolds
Source: Nat Commun. 2023 Dec 12;14:8253. doi: 10.1038/s41467-023-43948-y (PMC10716191; doi:10.1038/s41467-023-43948-y)
Supplement: Supplementary file 3 — Description of Additional Supplementary Files [file 41467_2023_43948_MOESM3_ESM.pdf]

Description of Additional Supplementary Files

Supplementary Data 1: Optimized structure of **Tol-Tpd-sNDI**

Supplementary Data 2: Optimized structure of **Tol-Tpd-nNDI**

Supplementary Movie 1: Calculated animation of vibrational mode at 199 cm^{-1} of **Tol-Tpd-nNDI**

Supplementary Movie 2: Calculated animation of vibrational mode at 500 cm^{-1} of **Tol-Tpd-nNDI**

Supplementary Movie 3: Calculated animation of vibrational mode at 750 cm^{-1} of **Tol-Tpd-nNDI**

Supplementary Movie 4: Calculated animation of vibrational mode at 1150 cm^{-1} of **Tol-Tpd-nNDI**

Supplementary Movie 5: Calculated animation of vibrational mode at 1655 cm^{-1} of **Tol-Tpd-nNDI**
